# Physicochemical cues are not potent regulators of human dermal fibroblast trans-differentiation^☆^

**DOI:** 10.1016/j.bbiosy.2023.100079

**Published:** 2023-05-29

**Authors:** Christina N.M. Ryan, Eugenia Pugliese, Naledi Shologu, Diana Gaspar, Peadar Rooney, Md Nahidul Islam, Alan O'Riordan, Manus J. Biggs, Matthew D. Griffin, Dimitrios I. Zeugolis

**Affiliations:** a Regenerative, Modular & Developmental Engineering Laboratory (REMODEL), Biomedical Sciences Building, University of Galway, Galway, Ireland; b Science Foundation Ireland (SFI) Centre for Research in Medical Devices (CÚRAM), Biomedical Sciences Building, University of Galway, Galway, Ireland; c Regenerative Medicine Institute (REMEDI), School of Medicine, Biomedical Sciences Building, University of Galway, Galway, Ireland; d Discipline of Biochemistry, School of Natural Sciences, University of Galway, Galway, Ireland; e Tyndall National Institute, University College Cork (UCC), Cork, Ireland; f Regenerative, Modular & Developmental Engineering Laboratory (REMODEL), Charles Institute of Dermatology, Conway Institute of Biomolecular & Biomedical Research and School of Mechanical & Materials Engineering, University College Dublin (UCD), Dublin, Ireland

**Keywords:** Collagen type I coating, Surface topography, Substrate rigidity, Macromolecular crowding, In vitro microenvironment, Fibroblast plasticity

## Abstract

Due to their inherent plasticity, dermal fibroblasts hold great promise in regenerative medicine. Although biological signals have been well-established as potent regulators of dermal fibroblast function, it is still unclear whether physiochemical cues can induce dermal fibroblast trans-differentiation. Herein, we evaluated the combined effect of surface topography, substrate rigidity, collagen type I coating and macromolecular crowding in human dermal fibroblast cultures. Our data indicate that tissue culture plastic and collagen type I coating increased cell proliferation and metabolic activity. None of the assessed in vitro microenvironment modulators affected cell viability. Anisotropic surface topography induced bidirectional cell morphology, especially on more rigid (1,000 kPa and 130 kPa) substrates. Macromolecular crowding increased various collagen types, but not fibronectin, deposition. Macromolecular crowding induced globular extracellular matrix deposition, independently of the properties of the substrate. At day 14 (longest time point assessed), macromolecular crowding downregulated tenascin C (in 9 out of the 14 groups), aggrecan (in 13 out of the 14 groups), osteonectin (in 13 out of the 14 groups), and collagen type I (in all groups). Overall, our data suggest that physicochemical cues (such surface topography, substrate rigidity, collagen coating and macromolecular crowding) are not as potent as biological signals in inducing dermal fibroblast trans-differentiation.

## Highlights

• Anisotropic surface topography induced bidirectional cell morphology on rigid substrates.
• Macromolecular crowding increased various collagen types, but not fibronectin, deposition.
• Physicochemical cues did not induce human dermal fibroblast trans-differentiation.

## Graphical abstract

The synergistic effect of surface topography, substrate rigidity, collagen type I coating and macromolecular crowding in human dermal fibroblast response.

**Figure:**
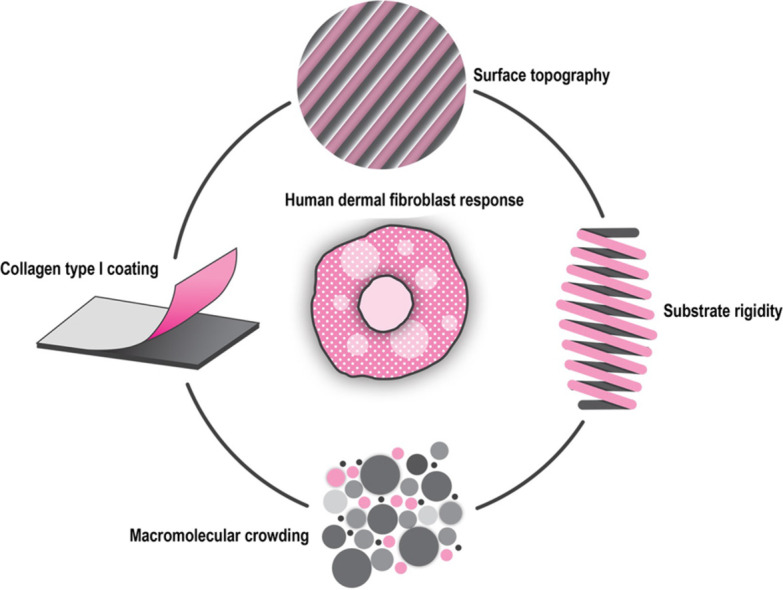

## 1 Introduction

The integrity of the adult human body depends on the phenotypic stability of cells within a tissue, whilst instability of differentiation that is associated with cellular trans-formation or trans-differentiation reflects phenotypic plasticity [1], [2], [3]. Fibroblasts are heterogeneous [4,5], mesenchymal in origin [6], [7], [8], [9], the most abundant cell type, the most efficient extracellular matrix (ECM) builders in the animal body and involved in both regenerative processes [10,11] and pathophysiological conditions [12,13]. Subject to how fibroblast will respond to specific stimuli, it will determine their function. For example, in vitro, following the discovery of cellular reprogramming [14,15], fibroblasts treated with appropriate cardiac, neuronal, chondrogenic, osteogenic or tenogenic factors can be reprogrammed into proliferative induced cardiac progenitor cells [16], induced neurons [17], induced chondrogenic cells [18], induced osteoblasts [19] or induced tenocytes [20], respectively. Despite the significant advancements in the field and the endless potential of induced pluripotent stem cells in drug discovery [21,22] and regenerative medicine [23,24], several scientific, social, legal and ethical limitations have arisen, restricting their use [25,26].

Biophysical stimuli have emerged as a safe and scalable approach to induce trans-differentiation of dermal fibroblasts. For example, high density cultures have been shown to induce tenogenic phenotype in human dermal fibroblasts cultures, albeit transient [27]. Dermal fibroblast within electrospun fibres under static mechanical strain have been shown to generate neo-tendon in vitro indistinguishable from the neo-tendon derived from human tenocytes [28]; unfortunately such approach requires prolonged in vitro culture (14 weeks) that is associated with high manufacturing costs and the optimal mechanical stimulation regime is still elusive [29]. To address these limitations, alternative approaches have been surfaced. For example, macromolecular crowding (MMC) has been shown to dramatically enhance and accelerate ECM deposition in both permanently differentiated and stem cell populations [30], [31], [32]. Surface topography [33], [34], [35], substrate rigidity [36], [37], [38], ECM / ligand tethering [39], [40], [41], alone or in combination (e.g. topography and rigidity [42,43], rigidity and ECM tethering [44,45]) have been shown to be potent regulators of cell function. Yet again, these in vitro microenvironment modulators have yet to be assessed in combination in fibroblast cultures. Thus, herein, we ventured to assess whether physicochemical in nature stimuli (i.e. MMC, surface topography, substrate rigidity, collagen type I coating) are capable in trans-differentiating human dermal fibroblasts in culture. We targeted musculoskeletal phenotypes, as previous publications have established the trans-differentiation potential of dermal fibroblasts towards tenogenic [46,47], chondrogenic [48], [49], [50] and osteogenic [51,52] lineages.

## 2 Materials and methods

### 2.1 Materials

All chemicals, cell culture media and reagents were purchased from Sigma Aldrich (Ireland), unless otherwise stated. Tissue culture consumables were purchased from Sarstedt (Ireland) and NUNC (Denmark).

### 2.2 Substrate fabrication

Polydimethylsiloxane (PDMS) substrates with different substrate rigidity, with and without anisotropic surface topography and with and without collagen type I coating were fabricated as has been described previously [53]. Briefly, a silicon wafer with groove dimensions of 2000 nm (groove depth) × 2000 nm (groove width) × 2000 nm (line width) was fabricated and thermal imprinting was used to transfer the pattern on the surface of the PDMS substrates with different rigidity (50 kPa, 130 kPa and 1000 kPa) that were fabricated by mixing appropriate ratios of Sylgard® 184 and Sylgard® 527 (Dow Corning, USA). The produced substrates were coated with 0.25 ml of 0.5 mg/ml in-house extracted bovine Achilles tendon collagen type I [54,55]. The dimensionality of the grooves of the substrates used is provided in **Table S1**.

### 2.3 Cell culture

Human dermal fibroblasts were purchased from LG Standards (UK) and used experimentally at passages 4–9. Cells were cultured in Dulbecco's Modified Eagle's Medium supplemented with 10% foetal bovine serum, and 1% penicillin/streptomycin and were maintained at 37 °C in a humidified atmosphere of 5% CO_2_. A density of 25,0000 cells/cm^2^ was seeded on the substrates. After 24 h, culture media were replaced with media containing 100 *µ*M ascorbic acid phosphate (for collagen synthesis [56], [57], [58], [59]) and 100 *µ*g/ml carrageenan (for collagen deposition [60], [61], [62], [63], [64]). Media were then changed every 3–4 days. Analysis was conducted at day 3, 7 and 14 from the first MMC supplementation.

### 2.4 Cell viability, metabolic activity and proliferation analyses

Cell viability was assessed with the Live/Dead® assay (Invitrogen, Ireland), following the manufacturer's instructions. Briefly, cells were washed with Hank's Balanced Salt Solution and a solution of 4 *µ*M calcein AM and 2 *µ*M ethidium homodimer I was added to each well. Following incubation at 37 ºC for 30 min, cells were imaged at 10x magnification, using an Olympus IX-81 (Japan) inverted fluorescence microscope.

Cell metabolic activity was assessed with the alamarBlue® assay (ThermoFisher Scientific, Ireland), following the manufacturer's instructions. In brief, cells were washed with Hank's Balanced Salt Solution, 10% alamarBlue® in Hank's Balanced Salt Solution was added to each well and the lates were incubated for 4 h at 37 ºC and 5% CO_2_. The absorbence of the solution was then measured at 550 nm and 595 nm with a Varioskan Flash Spectral scanning multimode reader (ThermoFisher Scientific, Ireland). Results were expressed as percentage reduction of the alamarBlue® dye and normalised to the tissue culture plastic (TCP) control.

Cell proliferation was assessed via cell number analysis. Cells were fixed with 4% paraformaldehyde, permeabilised with 0.2% Triton X-100 and nuclei were stained with 4′,6-diamidino-2-phenylindole (DAPI). Images were captured with an Olympus IX-81 inverted fluorescence microscope at 10x magnification. One substrate per technical triplicate was imaged and five fields-of-view were taken from each substrate. Fluorescent images were analysed using ImageJ (NIH, USA).

### 2.5 Cell morphometric analysis

Cells were fixed with 4% paraformaldehyde, permeabilised with 0.2% Triton X-100 and stained with DAPI (nuclei) and rhodamine labelled phalloidin (cytoskeleton). Using an Olympus IX-8 inverted fluorescence microscope, fluorescent images were acquired at 10x magnification. To determine nuclear area and aspect ratio (major axis / minor axis), one substrate per technical triplicate was imaged and five fields-of-view were taken from each substrate (in total, fifteen images analysed per experimental group). Images were analysed using ImageJ (NIH, USA).

### 2.6 Immunocytochemistry analysis

Cell layers were fixed with 4% paraformaldehyde, blocked with 3% serum albumin in phosphate buffered saline for 30 min, incubated with a primary antibody for 90 min, washed with phosphate buffered saline and incubated with a secondary antibody for 30 min (**Table S2** provides the details of the antibodies). Nuclei were counterstained with DAPI for 5 min. For quantification purposes, fluorescent area per image was quantified using ImageJ (NIH, USA) and normalised to cell number.

### 2.7 Fast Fourier transform analysis

To determine nuclei, cytoskeleton and deposited ECM orientation, 8-bit grey-scale images were taken from five fields-of-view of three stained substrates (in total, fifteen images were analysed per experimental group). Images were captured using an Olympus IX-81 inverted fluorescent microscope and processed with the fast Fourier transform function of ImageJ (NIH, USA). Fast Fourier transform images were rotated 90 ° right and radial intensity summing was performed and plotted in relation to angle of acquisition using the ImageJ plug-in ‘Oval Profile’.

### 2.8 Focal adhesion kinase analysis

Focal adhesion kinase (FAK) was measured at day 3 using the in vitro SimpleStep ELISA® kit, following the manufacturer's protocol (Abcam, UK). FAK standards (0–100 pg/ml) and samples were prepared and added to a pre-coated 96-well ELISA plate provided. The detection antibody was added and incubated for 3 h at room temperature on a plate shaker at 400 rpm. The plate was washed and the 3,3′,5,5′-tetramethylbenzidine substrate solution was added to each well and incubated for 10 min in the dark on a plate shaker at 400 rpm. The stop solution was then added to each well and the absorbence was measured at 450 nm with a Varioskan Flash Spectral scanning multimode reader (ThermoFisher Scientific, Ireland).

### 2.9 Gene expression analysis

For gene expression analysis, a TaqMan® RealTime ready Custom Panel for RT-qPCR (Roche, Ireland) was used. Briefly, total RNA was extracted from the cells after 7 and 14 days of culture using TRI Reagent® solution (Roche, Ireland). The Tri Reagent® was collected, chloroform was added and the solution was vortexed and centrifuged to recover the upper aqueous phase containing the RNA. This solution was mixed with ethanol and eluted through a column from the High Pure RNA isolation kit (Roche, Ireland). Only samples with an RNA integrity above 8, tested with the Agilent 2100 Bioanalyser (Agilent Technologies, Ireland), were further processed. Total RNA concentration was measured using the NanoDrop 1000 (ThermoFisher Scientific, Ireland) and 1 *µ*g of RNA sample was used for all the conditions. By means of a Transcriptor First Strand cDNA synthesis kit (Roche, Ireland), RNA was transcribed to cDNA. The thermal block cycle was set at 50 ºC for 1 h and at 85 ºC for 5 min for enzyme inactivation. A RealTime ready custom 384 well plate (Roche, Ireland) was run in the LightCycler® 480 Instrument (Roche, Ireland) to assess the expression of target genes (**Table S3** provides the details of the genes assessed). Using the 2-ΔCt method, mean Ct values of each target gene were normalised to the housekeeping gene values and the changes in gene expression between the TCP control and all other conditions at each time point were analysed using the 2-ΔΔCt method. Z-scores of fold changes were calculated and relevant up- and down- regulations were accepted when the score was at least two standard deviations away from the mean value of fold-change for each gene.

### 2.10 Statistical analysis

Biological analysis was conducted on three technical replicates and results are expressed as mean ± standard deviation. Statistical analysis was performed using SPSS® Version 24 (USA) and/or Prism Version 8 (USA). Multifactorial analysis of variance (ANOVA / F test) was used for multiple comparisons and a Student-Newman-Keuls post hoc test was used for pairwise comparisons if the samples followed a normal distribution (Kolmogorov-Smirnov test) and had equal variances (Bartlett's and Levene's test for homogeneity of variances). If these assumptions were violated, non-parametric tests were used for multiple comparisons (Kruskal-Wallis test / K test) and pairwise comparisons (Mann-Whitney U test / U test). Statistical significance was accepted at *p* < 0.05.

## 3 Results

### 3.1 Cell morphometric and FAK analyses

**Fig. 1:**
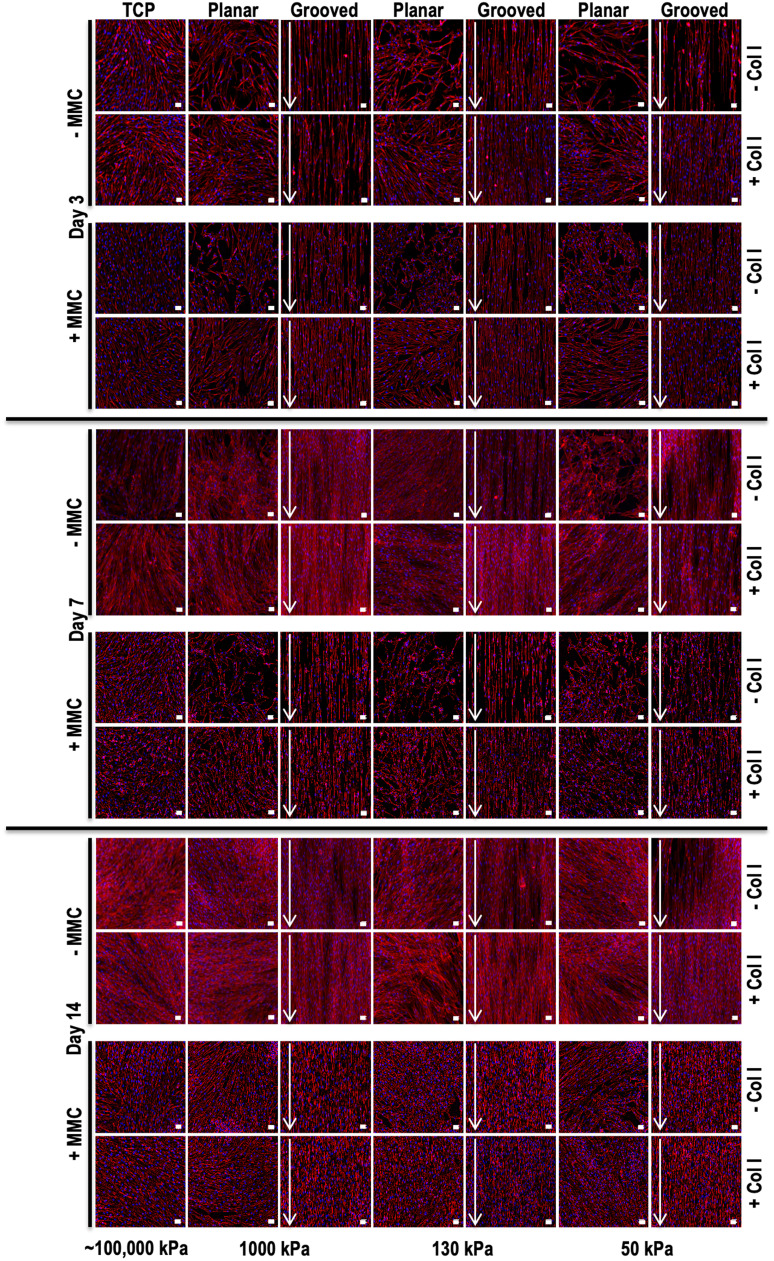
Human dermal fibroblast morphology at days 3, 7 and 14 on tissue culture plastic (TCP) without and with collagen type I coating (- Col, + Col) and macromolecular crowding (- MMC, + MMC) and on substrates of varying stiffness (1000 kPa, 130 kPa, 50 kPa), surface topography (planar, grooved), collagen type I coating (- Col, + Col) and macromolecular crowding (- MMC, + MMC), as shown using rhodamine phalloidin and DAPI staining. White arrow indicates direction of surface topography. Scale bar = 50 *μ*m.

All PDMS substrates and TCP promoted similar cell attachment and spreading, with the cells showing a random orientation on planar substrates and a highly aligned orientation, in the direction of the grooves, on grooved substrates, as confirmed by qualitative rhodamine phalloidin and DAPI staining analysis (Fig. 1). Quantitative cytoskeleton (**Figures S1-S3)** and nuclei (**Figures S4-S6)** orientation analyses revealed a cytoskeleton alignment in a singular peak at ∼ 90 º (less strict alignment on 50 kPa substrates and on 50 kPa substrates with MMC) and nuclei aligned in two major peaks at ∼ 70 º and ∼ 110 º. In general, the cells had significantly (*p* < 0.05) smaller nuclei on PDMS substrates, as opposed to TCP and on grooved, as opposed to on planar, substrates and collagen type I coating significantly (*p* < 0.05) increased nuclei area at day 3 and day 7, whilst MMC significantly (*p* < 0.05) decreased nuclei area at day 3 and day 7 and significantly (*p* < 0.05) increased nuclei area at day 14 (**Figure S7)**. Regarding nuclear elongation (**Figure S8)**, nuclei were significantly (*p* < 0.05) more elongated on grooved substrates at all time points, on collagen type I coated substrates at day 7 and 14 and under MMC conditions at day 14. FAK levels were significantly (*p* < 0.05) higher on TCP than on PDMS groups, higher on collagen type I coated substrates and lower under MMC conditions (**Figure S9**).

### 3.2 Basic cellular function analysis

Overall, a significantly (*p* < 0.05) higher cell number (**Figure S10**) and metabolic activity (**Figure S11**) were observed on TCP, as opposed to on PDMS substrates and on collagen coated substrates, whilst MMC significantly (*p* < 0.05) decreased both cell number and metabolic activity. Qualitative cell viability analysis (**Figure S12**) revealed no apparent differences (*p* > 0.05) between the groups (over 95% for all conditions).

### 3.3 Protein analysis

**Fig. 2:**
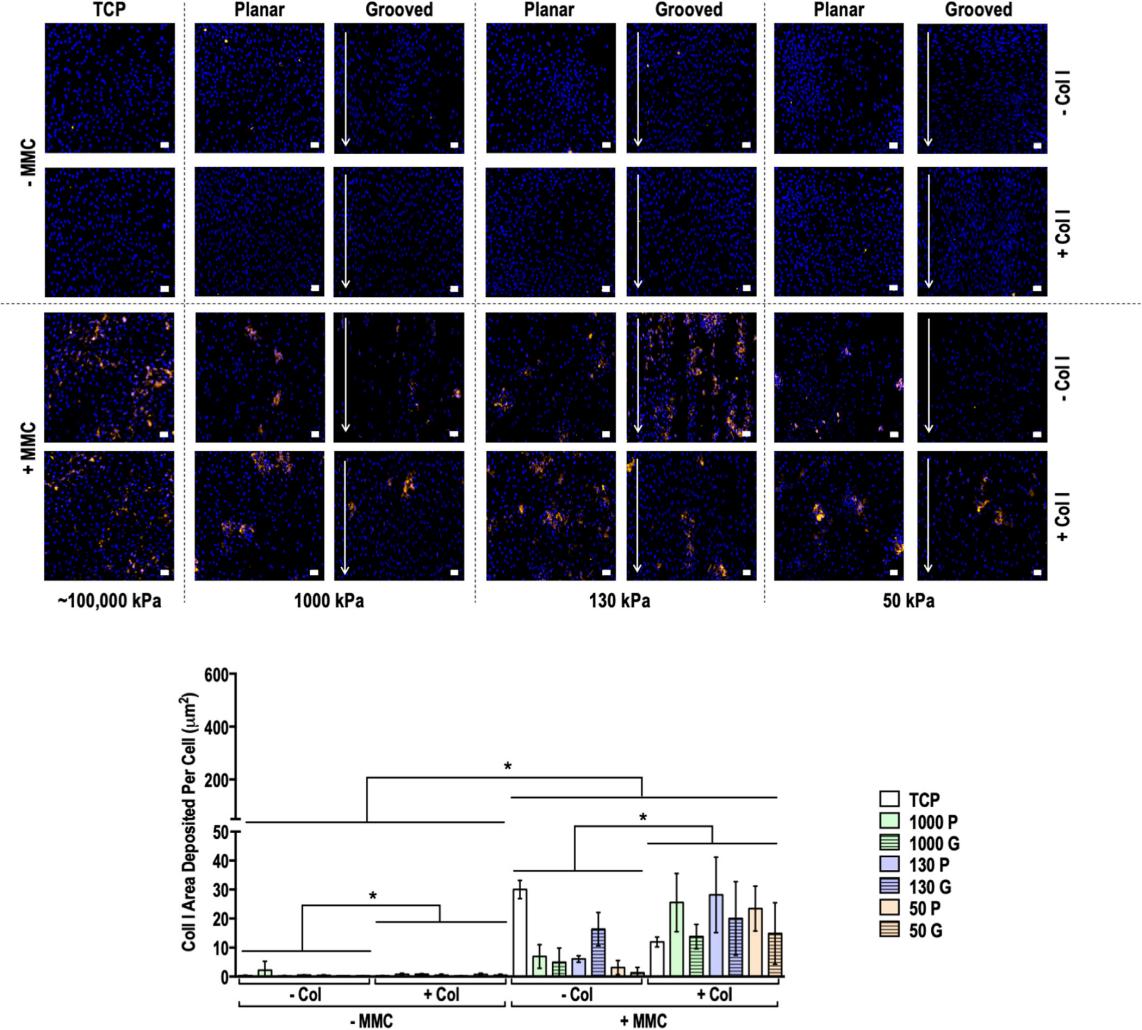
Human dermal fibroblast deposited collagen type I matrix and quantification of collagen type I matrix area deposited per cell at day 14 on tissue culture plastic (TCP) without and with collagen type I coating (- Col, + Col) and macromolecular crowding (- MMC, + MMC) and on substrates of varying stiffness (1000 kPa, 130 kPa, 50 kPa), surface topography [planar (P), grooved (G)], collagen type I coating (- Col, + Col) and macromolecular crowding (- MMC, + MMC). Collagen type I is represented in orange. DAPI is presented in blue. Scale bar = 50 *µ*m. * indicates statistically significant difference (*p* < 0.05) between without and with collagen type I coating and between without and with MMC.

**Fig. 3:**
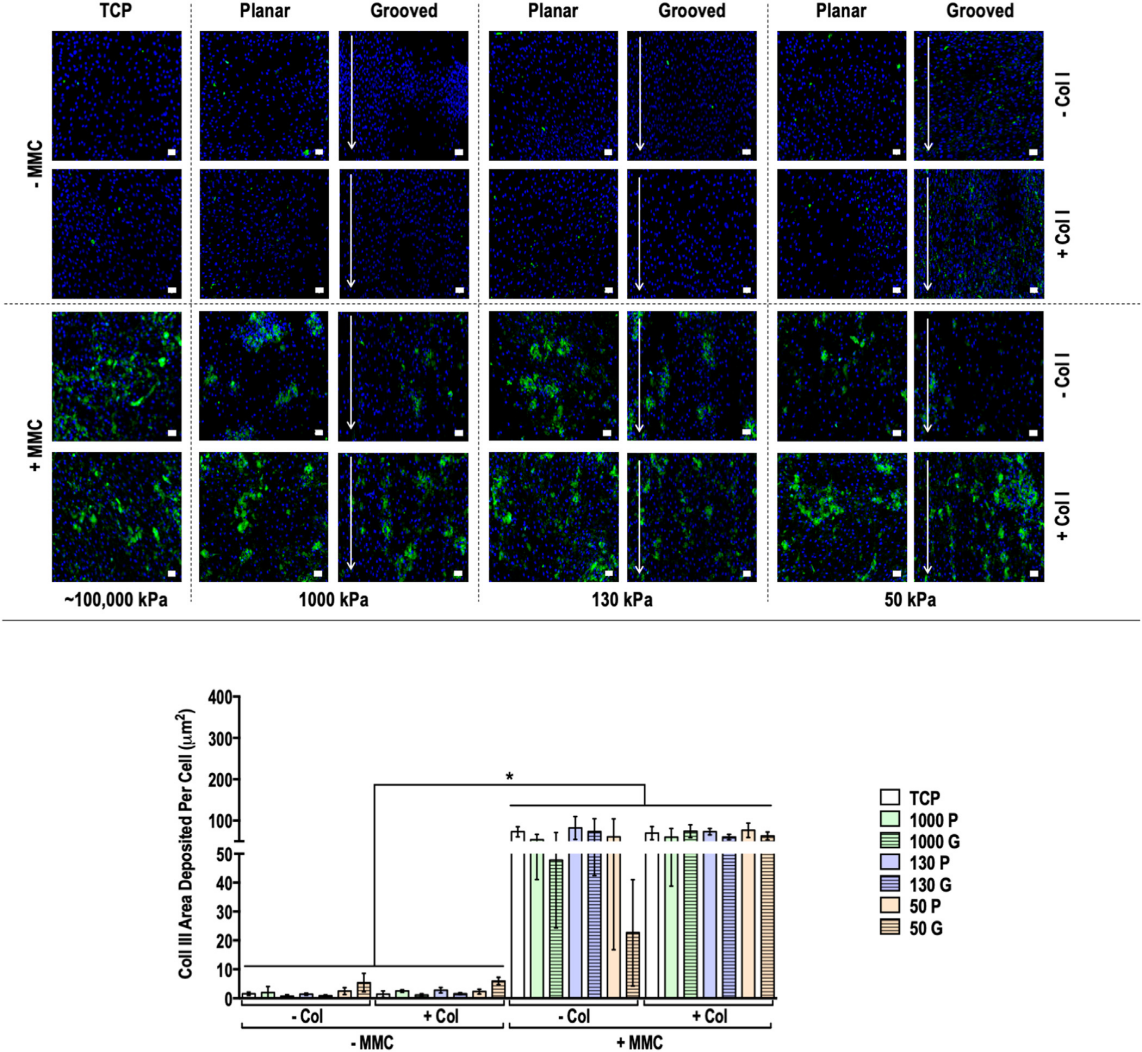
Human dermal fibroblast deposited collagen type III matrix and quantification of collagen type III matrix area deposited per cell at day 14 on tissue culture plastic (TCP) without and with collagen type I coating (- Col, + Col) and macromolecular crowding (- MMC, + MMC) and on substrates of varying stiffness (1000 kPa, 130 kPa, 50 kPa), surface topography [planar (P), grooved (G)], collagen type I coating (- Col, + Col) and macromolecular crowding (- MMC, + MMC). Collagen type III is represented in green. DAPI is represented in blue. Scale bar = 50 *µ*m. * indicates statistically significant difference (*p* < 0.05) between without and with MMC.

**Fig. 4:**
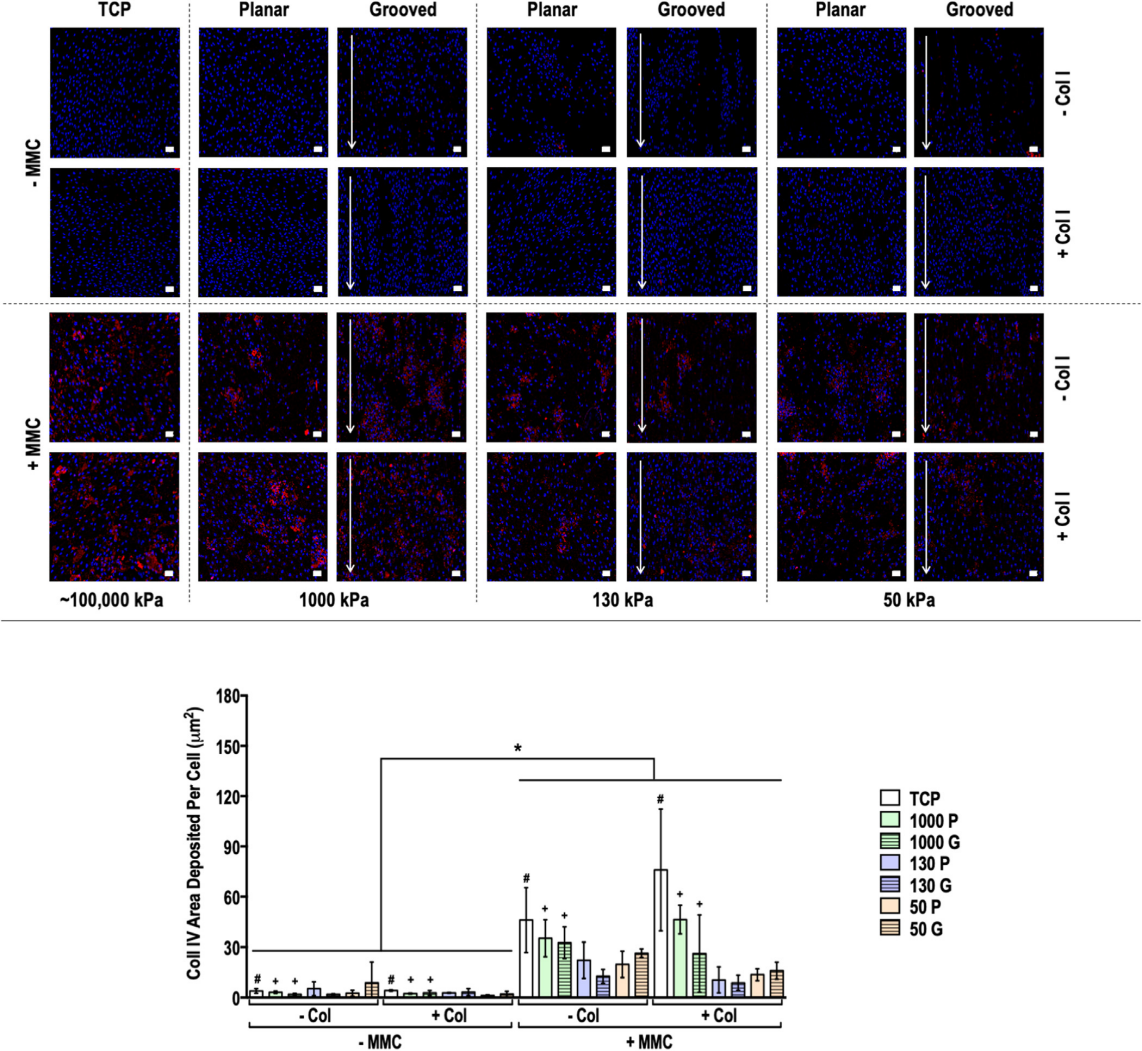
Human dermal fibroblast deposited collagen type IV matrix and quantification of collagen type IV matrix area deposited per cell at day 14 on tissue culture plastic (TCP) without and with collagen type I coating (- Col, + Col) and macromolecular crowding (- MMC, + MMC) and on substrates of varying stiffness (1000 kPa, 130 kPa, 50 kPa), surface topography [planar (P), grooved (G)], collagen type I coating (- Col, + Col) and macromolecular crowding (- MMC, + MMC). Collagen type IV is represented in red. DAPI is presented in blue. Scale bar = 50 *µ*m. * indicates statistically significant difference (*p* < 0.05) between without and with MMC, # indicates statistical difference (*p* < 0.05) between TCP and PDMS substrates and + indicates statistical difference (*p* < 0.05) between planar and grooved topography.

Immunocytochemistry and quantification of deposited matrix area analyses of collagen type I, collagen type III, collagen type IV, collagen type V, collagen type VI and fibronectin at day 3 (**Figures S13-S18**), day 7 (**Figures S19-S24**) and day 14 (Fig. 2, Fig. 3, Fig. 4 and **Figures S25-S27**) revealed that, in general, MMC treatment significantly (*p* < 0.05) increased collagen deposition and decreased fibronectin deposition. In general, surface topography, substrate rigidity and collagen type I coating did not significantly (*p* > 0.05) increase matrix deposition. Regarding ECM morphology (**Figures S28-S33)**, in the absence of MMC, there were no quantifiable amounts of collagen type I, collagen type III and collagen type IV at day 3, day 7 and day 14 for analysis. Overall, in the absence of MMC, the deposited matrix was aligned to the direction of the grooves, whilst in the presence of MMC, the deposited matrix appeared globular. In general, neither substrate rigidity nor collagen coating affected deposited matrix orientation.

### 3.4 Gene analysis

**Fig. 5:**
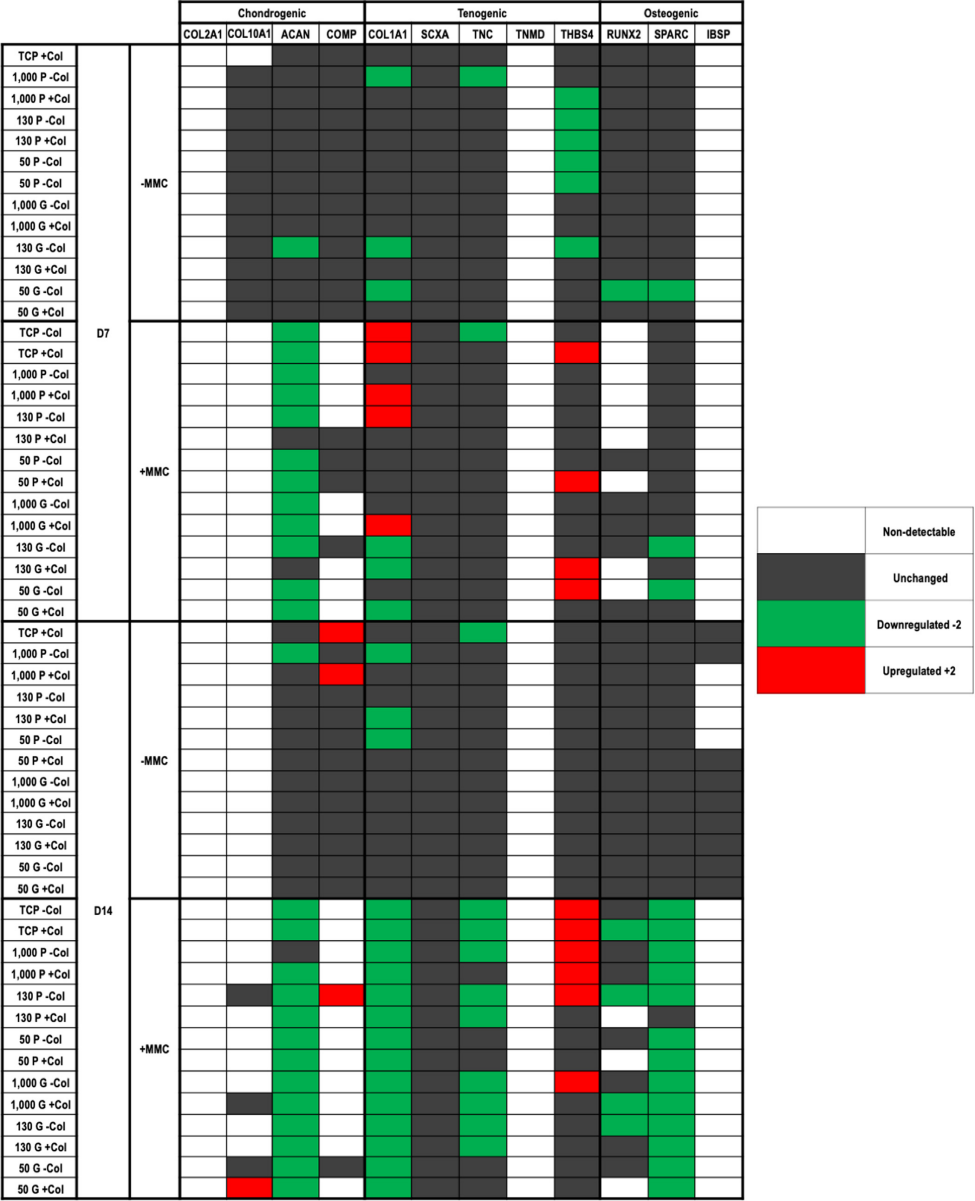
TaqMan® array showing human dermal fibroblast expression of chondrogenic, tenogenic and osteogenic genes on tissue culture plastic (TCP) without and with collagen type I coating (- Col, + Col) and macromolecular crowding (- MMC, + MMC) and on substrates of varying stiffness (1000 kPa, 130 kPa, 50 kPa), surface topography [planar (P), grooved (G)], collagen type I coating (- Col, + Col) and macromolecular crowding (- MMC, + MMC) at day 7 and day 14. Data are expressed in relation to TCP without collagen type I coating (- Col) and without macromolecular crowding (- MMC) at a given timepoint. White background: Not detectable. Grey background: Unchanged. Green background: Downregulated 2-fold. Red background: Upregulated 2-fold.

Gene expression analysis (Fig. 5) showed that at day 7 in the absence of MMC, the 130 kPa and the 50 kPa grooved substrates without collagen type I coating downregulated the most genes (ACAN, COL1A1, THBS4 and COL1A1, RUNX2, SPARC, respectively). At day 7 in the presence of MMC, all substrates, but the 130 kPa planar with collagen type I coating and the 130 kPa grooved with collagen type I coating, downregulated ACAN. At day 14 in the absence of MMC, the TCP with collagen type I coating upregulated COMP and downregulated TNC; the planar 1000 kPa without collagen type I coating downregulated ACAN and COL1A1; the planar 1000 kPa with collagen type I coating upregulated COMP; and the planar 130 kPa with collagen type I coating and the planar 50 kPa without collagen type I coating downregulated COL1A1. At day 14 in the presence of MMC, all substrates, but the planar 1000 kPa without collagen type I coating, downregulated ACAN; all substrates downregulated COL1A1; all substrates, but the planar 1000 kPa with collagen type I coating and the planar and grooved 50 kPa with and without collagen type I coating, downregulated TNC; all substrates, but the planar 130 kPa with collagen type I coating, downregulated SPARC; the TCP without and with collagen type I coating, the planar 1000 kPa without and with collagen type I coating, the planar 130 kPa without collagen type I coating and the grooved 1000 kPa without collagen type I coating upregulated THBS4. COL2A1 and TNMD was not detected at all.

## 4 Discussions

Dermal fibroblasts hold a tremendous potential in regenerative medicine. Yet again, the influence of physicochemical cues in their function has yet to be assessed in a systematic fashion. Herein, we assessed the combined effect of surface topography, substrate rigidity, collagen type I coating and MMC on dermal fibroblast morphology, basic function, protein synthesis and gene expression.

### 4.1 Cell morphometric and FAK analyses

The cells attached and spread homogeneously on all planar substrates, whilst on grooved substrates, they aligned in the orientation of the grooves, as, due to contact guidance, cells follow the groove edge and do not cross the gap [65], [66], [67], [68], [69], [70], [71]. Interestingly, cells aligned less strictly on the softest substrates and on all substrates at the later time point, especially when MMC was used. Both these observation can be attributed to the reduction in groove height, as has been seen before [70], [71], [72], and/or the reduced rigidity, considering that substrate rigidity drives actin organisation and cell polarity [73,74]. Similarly to cytoskeleton, nuclei also aligned in the direction of the grooves, but with two major peaks (as opposed to one that was observed for the cytoskeleton), suggestive of reduced nuclei compliance to physical cues [75]. The morphological changes in both cytoskeleton and nuclei directly align with the mechano-transduction theory, which suggests that the intracellular tension in elongated and aligned cytoskeleton actin filaments is transferred to the nucleus through the cytoskeleton [76,77]. Collagen type I coating and MMC induced elongated nuclei morphology, especially on late time points, which can be attributed to the role of surface proteins [78,79] and/or substrate stiffness [80], [81], [82] on cell attachment and spreading. TCP induced the highest FAL levels, which can be attributed to rigidity difference; indeed, high rigidity has been shown to increase focal adhesion and FAK levels [83], [84], [85].

### 4.2 Basic cellular function analysis

Cell number, metabolic activity and viability analyses showed that, in general, the cells grew and proliferated well on all tested substrates / conditions. The very stiff TCP resulted in higher than PDMS cell number and metabolic activity, as has also been reported previously [86]. Collagen type I coating increased cell number and metabolic activity, which is linked to the role of the ECM proteins on cell attachment and proliferation [40,87]. Grooved substrates did not affect basic cellular functions, indicating that, although a similar bidirectional cell elongation can be achieved by topographical cues and mechanical stretching, only mechanical loading, in excessive form, can activate apoptotic pathways [88,89]. When cells were cultured in the presence of MMC, although lower cell number and metabolic activity values were observed, cell viability was above 95%, as with every other condition. It is worth noting that carrageenan has been used extensively as MMC agent in stem [60], [61], [62], [63], [64] and permanently differentiated [90], [91], [92], [93], [94], [95] cell cultures and in preclinical setting [96] without any negative effects.

### 4.3 Protein analysis

Substrate rigidity, surface topography and collagen type I coating had negligible effect on ECM deposition, whilst MMC substantially increased collagen deposition. According to the excluded volume effect theory, in crowded cultures, the diffusion of procollagen and proteinases is restricted, enabling the accelerated enzymatic processing of water-soluble pro-collagen to water-insoluble collagen [30], [31], [32]. On the other hand, MMC did not increase fibronectin deposition, which is not surprising, considering that fibronectin is not enzymatically processed [97]. Regarding the morphology of the assessed proteins, a more aligned structure was observed when the cells were cultured in the absence of MMC, whilst a rather globular morphology was apparent, when the cells were cultured under MMC conditions. We believe that the cells were not capable to process in an aligned fashion the vast amounts of deposited ECM. Possibly, larger in groove dimensionality substrates (e.g. 10 *μ*m wide and 3 *μ*m deep [98], 9 *μ*m wide and 1–2 *μ*m deep [99]) may be able to both induce cellular and deposited ECM alignment along the direction of the grooves. Rotating electrospinning can also be used to induce bidirectional cell and deposited ECM alignment [28,100], even under MMC conditions [101].

### 4.4 Gene analysis

Gene expression analysis made apparent that at both time points COL2A1 [102], TNMD [103,104] and IBSP [105], [106], [107] that are frequently associated with chondrogenesis, tenogenesis and osteogenesis, respectively, were not detected at all and MMC appeared to have an influence on cell phenotype. In particular, at day 14 (the longest time point assessed), downregulation of ACAN (in 13 out of the 14 groups), SPARC (in 13 out of the 14 groups), TNC (in 9 out of the 14 groups) and COL1A1 (in all groups) was observed in the MMC groups. Upregulation of ACAN [108,109], SPARC [110], [111], [112], [113] and TNC [114], [115], [116] is frequently associated with chondrogenesis, osteogenesis and fibrosis, respectively; their downregulation therefore is suggestive of physiological cellular phenotype. The downregulation of COL1A1 is attributed to the negative feedback loop effect, during which accumulation of a protein induces cells to suppress the expression of the corresponding gene [117], [118], [119]. Overall, the well documented plasticity of dermal fibroblasts [120], [121], [122], [123] was not verified herein, suggesting that the biophysical cues assessed are not of sufficient potency to drive human dermal fibroblast trans-differentiation.

## 5 Conclusions

The plasticity of dermal fibroblasts has been well-established in the literature, with their fate to depend upon the used in vitro microenvironment modulators. Considering that physicochemical signals are master regulators of cell fate and relatively easy in implementation, herein, we evaluated the influence of surface topography, substrate rigidity, collagen type I coating and MMC in human dermal fibroblast cultures. High rigidity and collagen type I coating increased cell proliferation and metabolic activity, whilst none of the assessed conditions affected cell viability. Surface topography was found to be a potent regulator of cell morphology, especially on more rigid substrates. Immunocytochemistry analysis made apparent that MMC substantially increased collagen, but not fibronectin, deposition, albeit in a globular fashion, even on the grooved substrates. Based on gene expression analysis data, a clear trans-differentiation towards a musculoskeletal-related lineage (i.e. tenogenic, chondrogenic and osteogenic) was not observed, independently of the conditions used. Collectively our data suggest that although physicochemical cues have a clear effect on cell function, they are not as potent as biological factors in dermal fibroblast trans-differentiation.

## Author contributions

DIZ had the overall responsibility of the work. DIZ and CNMR designed the study. CNMR performed brightfield and fluorescence microscopy analyses; optical profilometry analysis; cell viability and metabolic activity assays; immunocytochemistry and ImageJ analyses. CNMR and NS performed the cell culture experiments. CNMR, PR and DG performed gene expression experiments and measurements. CNMR and PR performed focal adhesion kinase measurements and analysis. NS and NI performed flow cytometry experiments and measurements. CNMR processed all the experimental data, performed the statistical analysis and designed the figures. MJB was involved in the fabrication of substrates with different rigidity used to culture the cells. AO'R performed the silicon wafer fabrication used to fabricate the grooved PDMS substrates. CNMR wrote the first draft of the manuscript and DIZ and EP edited and finalised manuscript and figures. All authors discussed and approved the final version of the manuscript.

## Declaration of Competing Interest

The authors have no competing interests to declare.

## Data Availability

The raw data required to reproduce these findings are available on request from CNMR. The processed data required to reproduce these findings are available on request from CNMR.

## Appendix Supplementary materials

Image, application 1
