# Supplementary material for: Physicochemical cues are not potent regulators of human dermal fibroblast trans-differentiation
Source: Biomater Biosyst. 2023 May 29;11:100079. doi: 10.1016/j.bbiosy.2023.100079 (PMC10499661; doi:10.1016/j.bbiosy.2023.100079)

# Supplementary Information

# Title

Physicochemical cues are not potent regulators of human dermal fibroblast trans-differentiation

# Authors

Christina N. M. Ryan (1, 2), Eugenia Pugliese (1, 2), Naledi Shologu (1, 2), Diana Gaspar (1, 2), Peadar Rooney (2), Md Nahidul Islam (2, 3, 4), Alan O’Riordan (5), Manus J. Biggs (2), Matthew D. Griffin (2, 3), Dimitrios I. Zeugolis* (1, 2, 6)

# Affiliations

(1) Regenerative, Modular & Developmental Engineering Laboratory (REMODEL), Biomedical Sciences Building, University of Galway, Galway, Ireland

(2) Science Foundation Ireland (SFI) Centre for Research in Medical Devices (CÚRAM), Biomedical Sciences Building, University of Galway, Galway, Ireland

(3) Regenerative Medicine Institute (REMEDI), School of Medicine, Biomedical Sciences Building, University of Galway, Galway, Ireland

(4) Discipline of Biochemistry, School of Natural Sciences, University of Galway, Galway, Ireland

(5) Tyndall National Institute, University College Cork (UCC), Cork, Ireland

(6) Regenerative, Modular & Developmental Engineering Laboratory (REMODEL), Charles Institute of Dermatology, Conway Institute of Biomolecular & Biomedical Research and School of Mechanical & Materials Engineering, University College Dublin (UCD), Dublin, Ireland

* Corresponding Author: Dimitrios I. Zeugolis, REMODEL, UCD. Telephone: +353 17 16 18 87; Email: dimitrios.zevgolis@ucd.ie

**Table S1:** Groove dimensionality of the used substrates.

| **PDMS substrate stiffness** | **Collagen type I coating (mg/ml)** | **Groove depth (nm)** | **Groove width (nm)** | **Line width (nm)** |
| --- | --- | --- | --- | --- |
| **1,000 kPa** | 0 | 1,987 ± 88 | 2,114 ± 435 | 1,439 ± 130 |
|  | 0.5 | 2,022 ± 102 | 2,180 ± 115 | 1,347 ± 156 |
| **130 kPa** | 0 | 1,938 ± 283 | 1,746 ± 152 | 1,917 ± 231 |
|  | 0.5 | 1,947 ± 87 | 2,319 ± 138 | 1,367 ± 77 |
| **50 kPa** | 0 | 1,855 ± 186 | 1,697 ± 112 | 1,787 ± 202 |
|  | 0.5 | 1,468 ± 219 | 2,835 ± 1,113 | 1,276 ± 138 |

**Table S2:** Primary and secondary antibodies used for immunocytochemistry analysis.

| **Protein Target** | **Primary Antibody** | **Company** | **Dilution** | **Secondary Antibody** | **Company** | **Dilution** |
| --- | --- | --- | --- | --- | --- | --- |
| **Collagen I** | Mouse (ab90395) | Abcam (UK) | 1 to 200 | Goat anti-mouse AlexaFluor® 555 (A-21422) | ThermoFisher Scientific (Ireland) | 1 to 500 |
| **Collagen III** | Rabbit (ab7778) | Abcam (UK) | 1 to 200 | Goat anti-rabbit AlexaFluor® 488 (A11034) | Life Technologies (UK) | 1 to 500 |
| **Collagen IV** | Rabbit (ab6586) | Abcam (UK) | 1 to 200 | Goat anti-rabbit AlexaFluor® 488 (A11034) | Life Technologies (UK) | 1 to 500 |
| **Collagen V** | Rabbit (ab7046) | Abcam (UK) | 1 to 200 | Donkey anti-rabbit AlexaFluor® 555 (ab150074) | Abcam (UK) | 1 to 500 |
| **Collagen VI** | Rabbit (ab6588) | Abcam (UK) | 1 to 200 | Goat anti-rabbit AlexaFluor® 488 (A11034) | Life Technologies (UK) | 1 to 500 |
| **Fibronectin** | Rabbit (ab2413) | Abcam (UK) | 1 to 200 | Donkey anti-rabbit AlexaFluor® 555 (ab150074) | Abcam (UK) | 1 to 500 |

**Table S3:** Primers used in RT-qPCR TaqMan® RealTime ready Custom Panel.

| **Lineage** | **Gene Name** | **Gene Symbol** | **Forward Primer (5' to 3')** | **Reverse Primer (5' to 3')** |
| --- | --- | --- | --- | --- |
| **Tenogenic** | Collagen type I α1 chain | COL1A1 | AGGTGAAGCAGGCAAACCT | CTCGCCAGGGAAACCTCT |
|  | Scleraxis homolog A | SCXA | CCCAAACAGATCTGCACCTT | TCTTTCTGTCGCGGTCCTT |
|  | Tenascin C | TNC | CCTTGCTGTAGAGGTCGTCA | CCAACCTCAGACACGGCTA |
|  | Tenomodulin | TNMD | TGTATTGGATCAATCCCACTCTAA | TCGTTGGCAGGAAAGTGAA |
|  | Thrombospondin 4 | THBS4 | CTACCGCTGGTTCCTACAGC | GAGCCTTCATAAAATCGTACCC |
| **Osteogenic** | Runt-related transcription factor | RUNX2 | GCCTAGGCGCATTTCAGAT | CTGAGAGTGGAAGGCCAGAG |
|  | Osteonectin | SPARC | TTGATGATGGTGCAGAGGAA | CTTGCCGTGTTTGCAGTG |
|  | Bone sialoprotein | IBSP | CAGGGCAGTAGTGACTCATCC | TCGATTCTTCATTGTTTTCTCCT |
| **Chondrogenic** | Collagen II | COL2A1 | CTGGTCCTCAAGGCAAAGTT | GAGGTCCAGGACGACCATC |
|  | Collagen X | COL10A1 | CAGTTCTTCATTCCCTACACCA | AGGACTTCCGTAGCCTGGTT |
|  | Aggrecan | ACAN | GAACGACAGGACCATCGAA | AAAGTTGTCAGGCTGGTTGG |
|  | Cartilage oligomeric protein | COMP | GGAGATCGTGCAGACAATGA | GTCATCCGTGACCGTGTTC |
| **Housekeeping** | Glyceraldehyde-3-phosphate dehydrogenase | GAPDH | AGCCACATCGCTCAGACAC | GCCCAATACGACCAAATCC |

**Figure S1:** Human dermal fibroblast cytoskeleton orientation at day 3 on tissue culture plastic (TCP) without and with collagen type I coating (- Col, + Col) and macromolecular crowding (- MMC, + MMC) and on substrates of varying stiffness (1,000 kPa, 130 kPa, 50 kPa), surface topography [planar (P), grooved (G)], collagen type I coating (- Col, + Col) and macromolecular crowding (- MMC, + MMC).


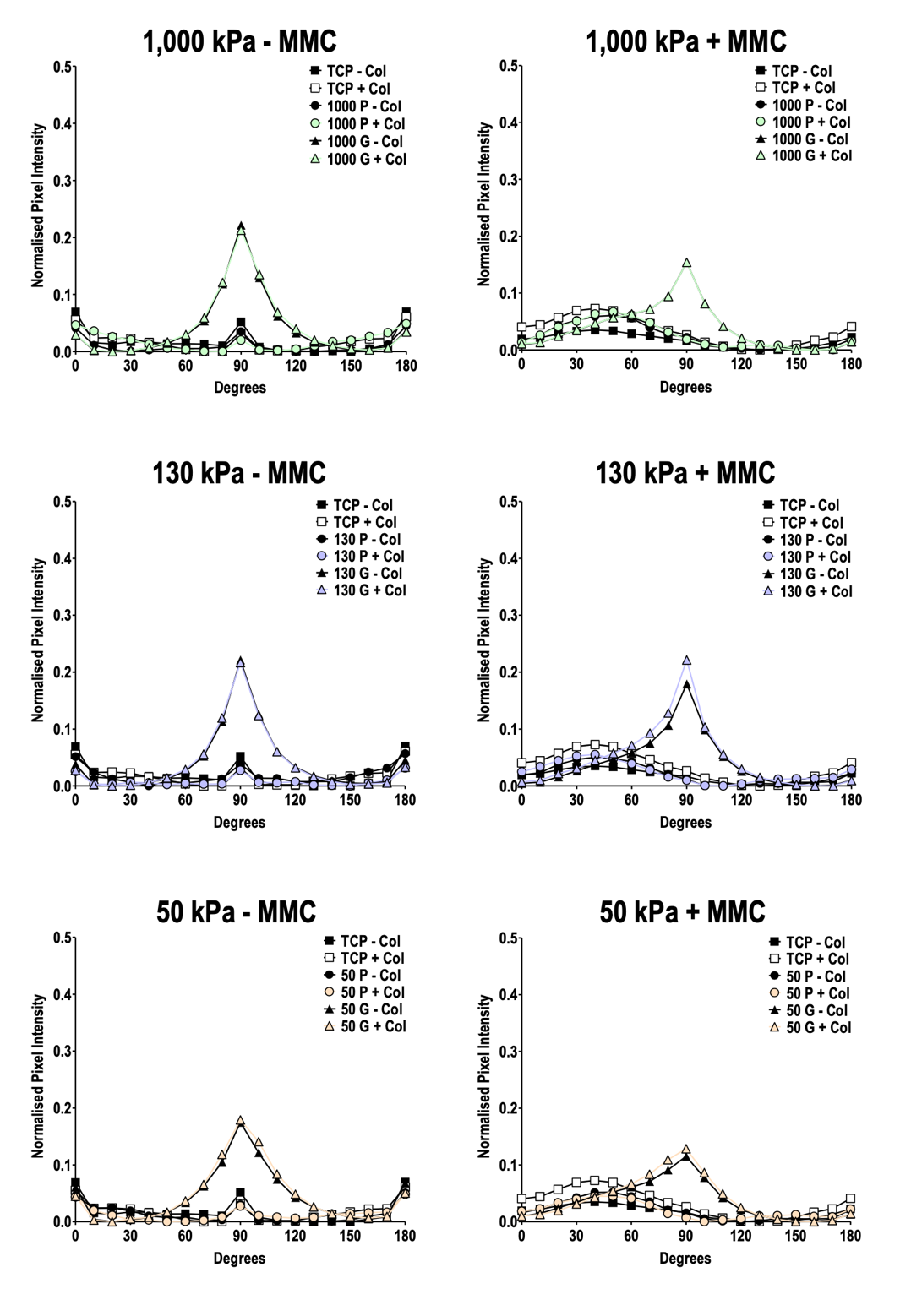


**Figure S2:** Human dermal fibroblast cytoskeleton orientation at day 7 on tissue culture plastic (TCP) without and with collagen type I coating (- Col, + Col) and macromolecular crowding (- MMC, + MMC) and on substrates of varying stiffness (1,000 kPa, 130 kPa, 50 kPa), surface topography [planar (P), grooved (G)], collagen type I coating (- Col, + Col) and macromolecular crowding (- MMC, + MMC).


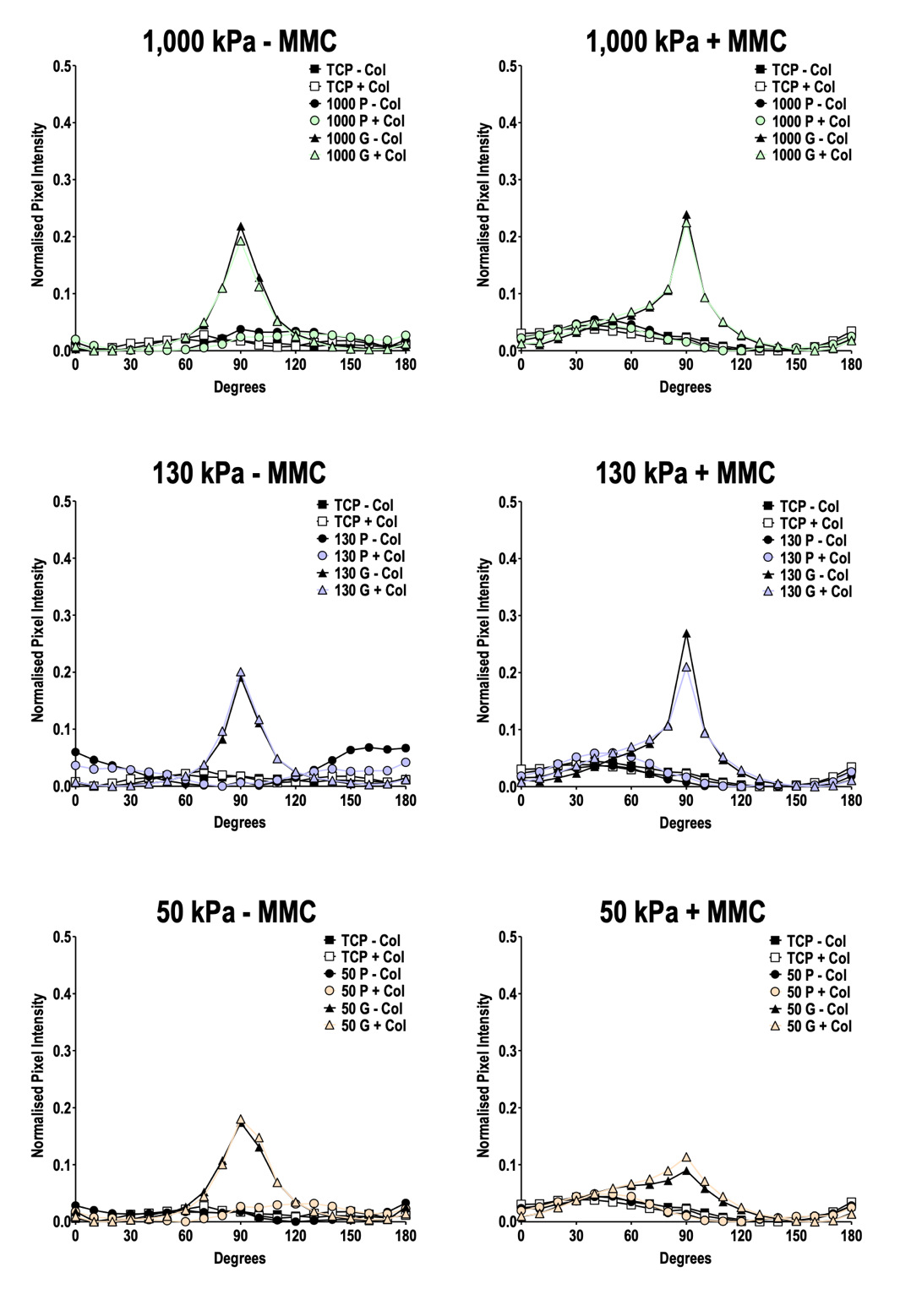


**Figure S3:** Human dermal fibroblast cytoskeleton orientation at day 14 on tissue culture plastic (TCP) without and with collagen type I coating (- Col, + Col) and macromolecular crowding (- MMC, + MMC) and on substrates of varying stiffness (1,000 kPa, 130 kPa, 50 kPa), surface topography [planar (P), grooved (G)], collagen type I coating (- Col, + Col) and macromolecular crowding (- MMC, + MMC).


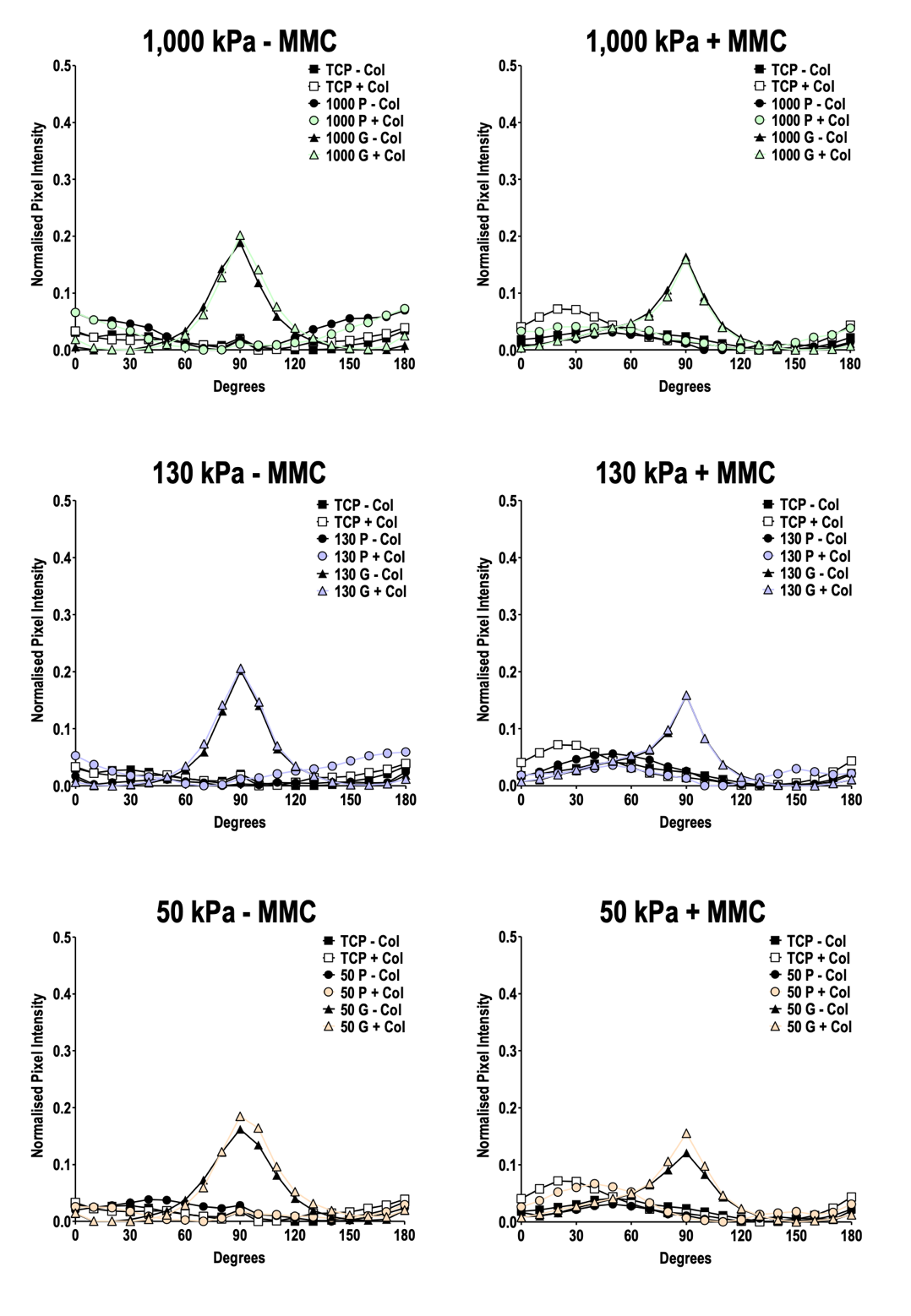


**Figure S4:** Human dermal fibroblast nuclear orientation at day 3 on tissue culture plastic (TCP) without and with collagen type I coating (- Col, + Col) and macromolecular crowding (- MMC, + MMC) and on substrates of varying stiffness (1,000 kPa, 130 kPa, 50 kPa), surface topography [planar (P), grooved (G)], collagen type I coating (- Col, + Col) and macromolecular crowding (- MMC, + MMC).


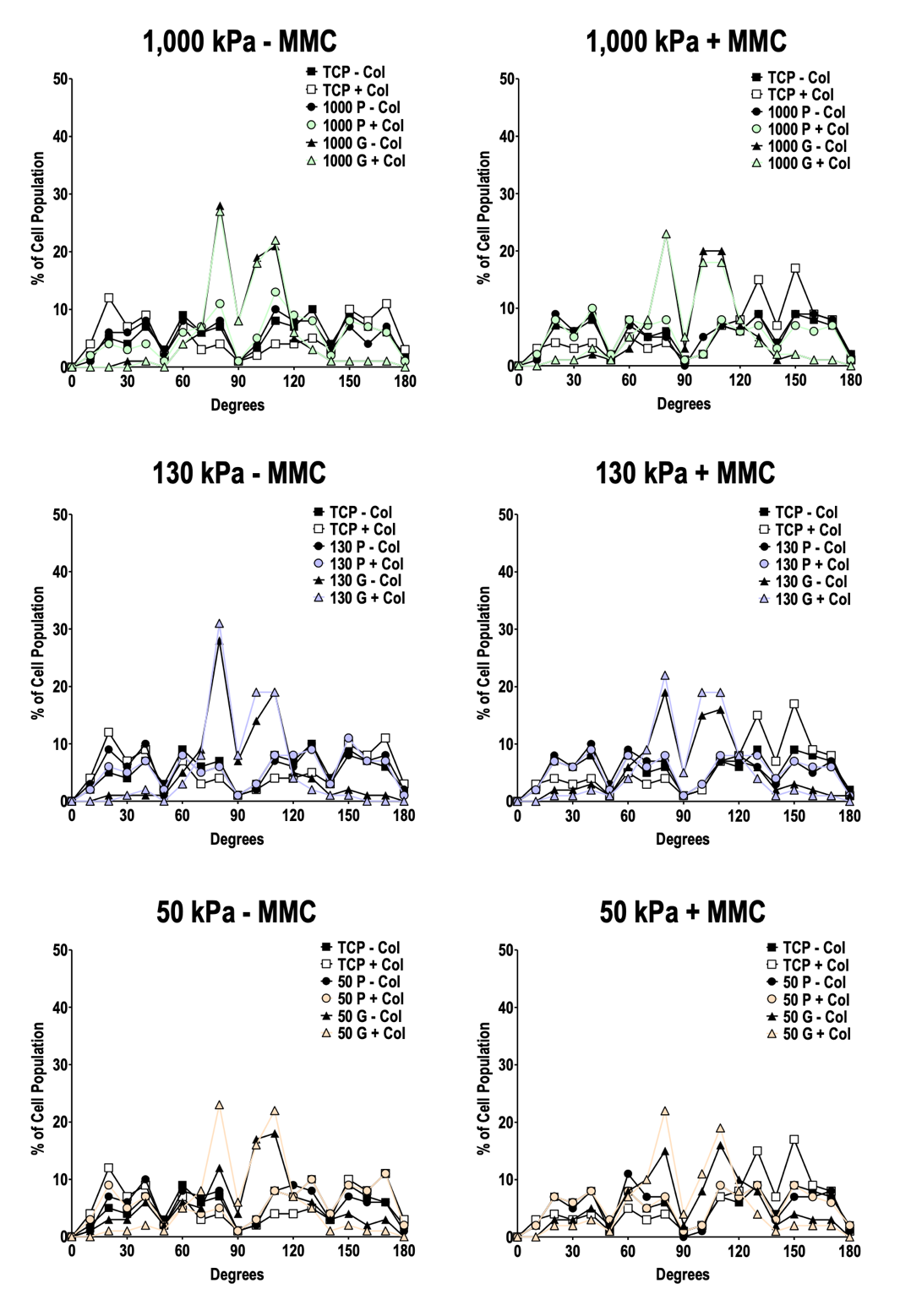


**Figure S5:** Human dermal fibroblast nuclear orientation at day 7 on tissue culture plastic (TCP) without and with collagen type I coating (- Col, + Col) and macromolecular crowding (- MMC, + MMC) and on substrates of varying stiffness (1,000 kPa, 130 kPa, 50 kPa), surface topography [planar (P), grooved (G)], collagen type I coating (- Col, + Col) and macromolecular crowding (- MMC, + MMC).


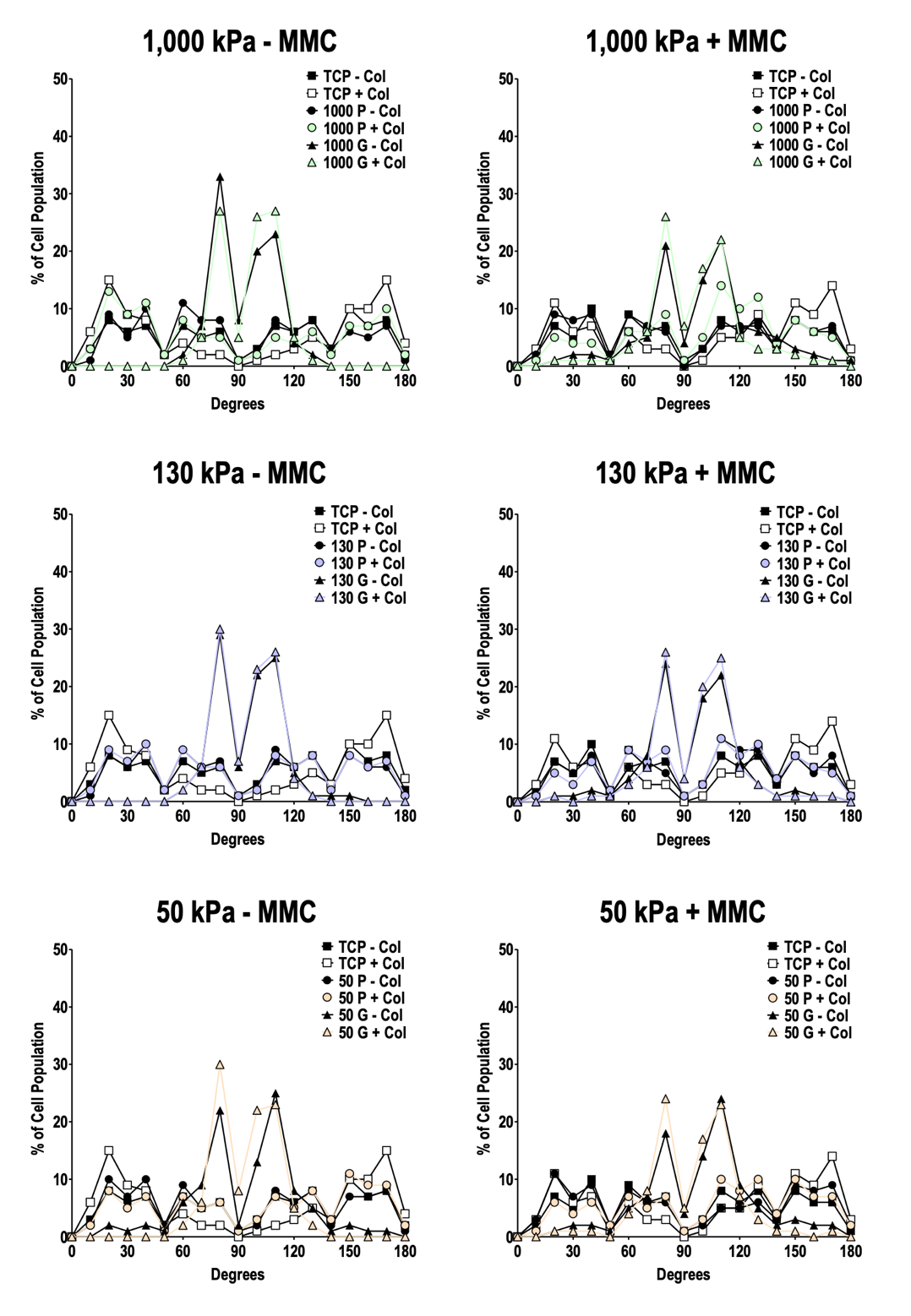


**Figure S6:** Human dermal fibroblast nuclear orientation at day 14 on tissue culture plastic (TCP) without and with collagen type I coating (- Col, + Col) and macromolecular crowding (- MMC, + MMC) and on substrates of varying stiffness (1,000 kPa, 130 kPa, 50 kPa), surface topography [planar (P), grooved (G)], collagen type I coating (- Col, + Col) and macromolecular crowding (- MMC, + MMC).


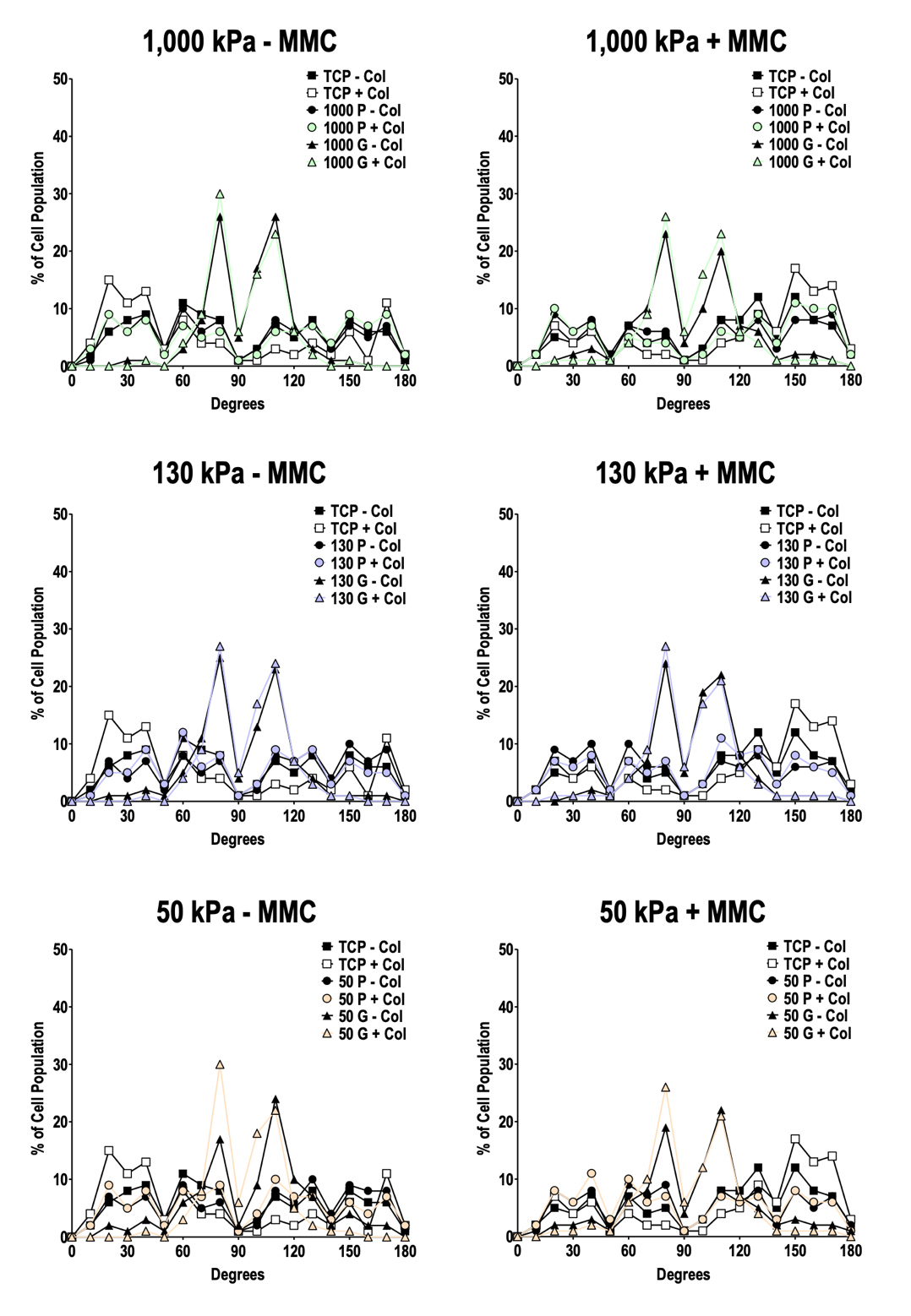


**Figure S7:** Human dermal fibroblast nuclear area of cells on tissue culture plastic (TCP) without and with collagen type I coating (- Col, + Col) and macromolecular crowding (- MMC, + MMC) and on substrates of varying stiffness (1,000 kPa, 130 kPa, 50 kPa), surface topography [planar (P), grooved (G)], collagen type I coating (- Col, + Col) and macromolecular crowding (- MMC, + MMC) at day 3, day 7 and day 14. * indicates statistically significant difference (p < 0.05) between without and with collagen type I coating and between without and with MMC, # indicates statistical difference (p < 0.05) between TCP and PDMS substrates and + indicates statistical difference (p < 0.05) between planar and grooved topography.


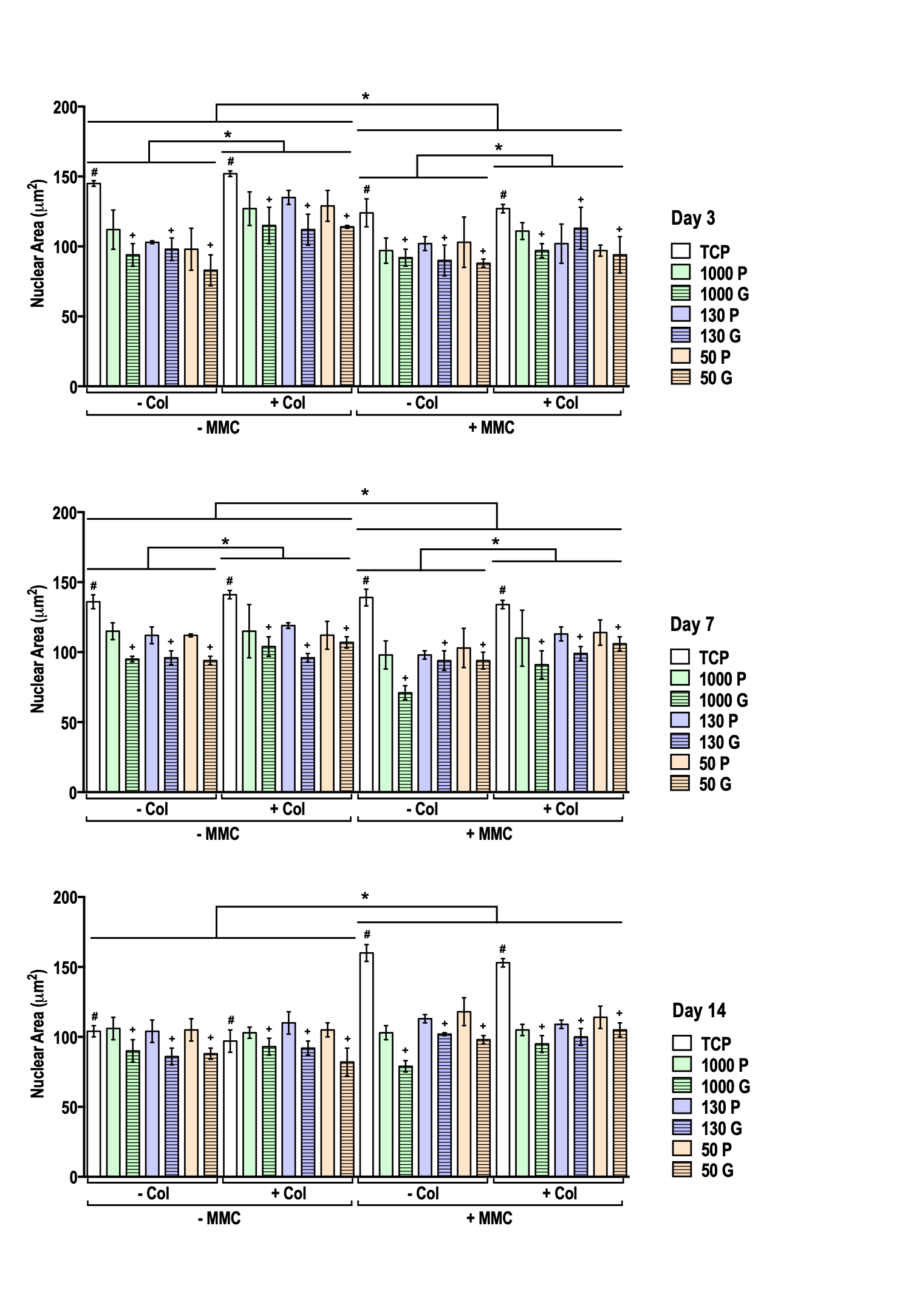


**Figure S8:** Human dermal fibroblast nuclear elongation of cells on tissue culture plastic (TCP) without and with collagen type I coating (- Col, + Col) and macromolecular crowding (- MMC, + MMC) and on substrates of varying stiffness (1,000 kPa, 130 kPa, 50 kPa), surface topography [planar (P), grooved (G)], collagen type I coating (- Col, + Col) and macromolecular crowding (- MMC, + MMC) at day 3, day 7 and day 14. * indicates statistically significant difference (*p* < 0.05) between without and with collagen type I coating and between without and with MMC, # indicates statistical difference (*p* < 0.05) between TCP and PDMS substrates and + indicates statistical difference (*p* < 0.05) between planar and grooved topography.


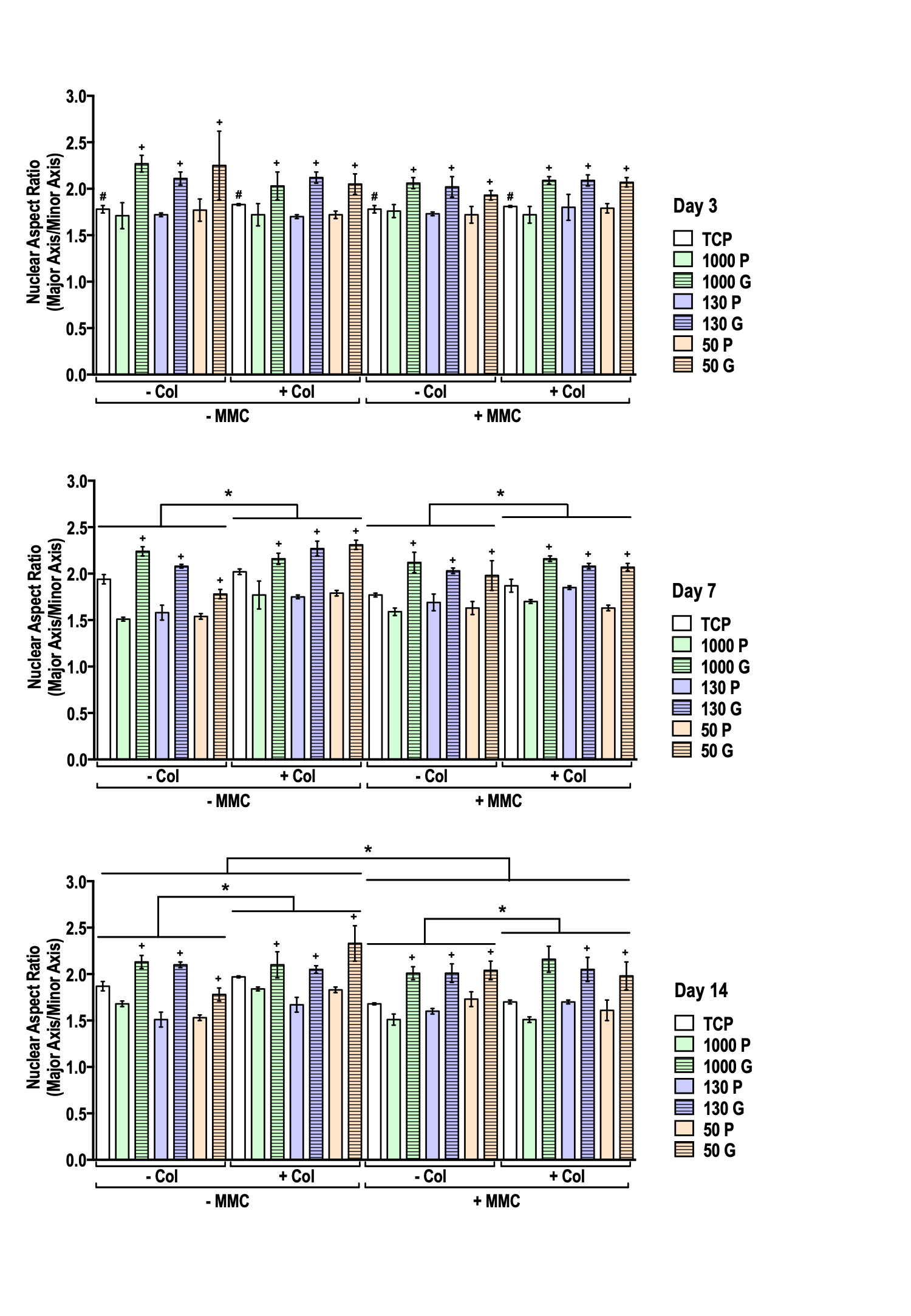


**Figure S9:** % FAK of total protein from human dermal fibroblasts cultured on tissue culture plastic (TCP) without and with collagen type I coating (- Col, + Col) and macromolecular crowding (- MMC, + MMC) and on substrates of varying stiffness (1,000 kPa, 130 kPa, 50 kPa), surface topography [planar (P), grooved (G)], collagen type I coating (- Col, + Col) and macromolecular crowding (- MMC, + MMC) at day 3. ND indicates values obtained below the detection limits of ELISA. * indicates statistically significant difference (*p* < 0.05) between without and with collagen type I coating and # indicates statistical difference (*p* < 0.05) between TCP and PDMS substrates.


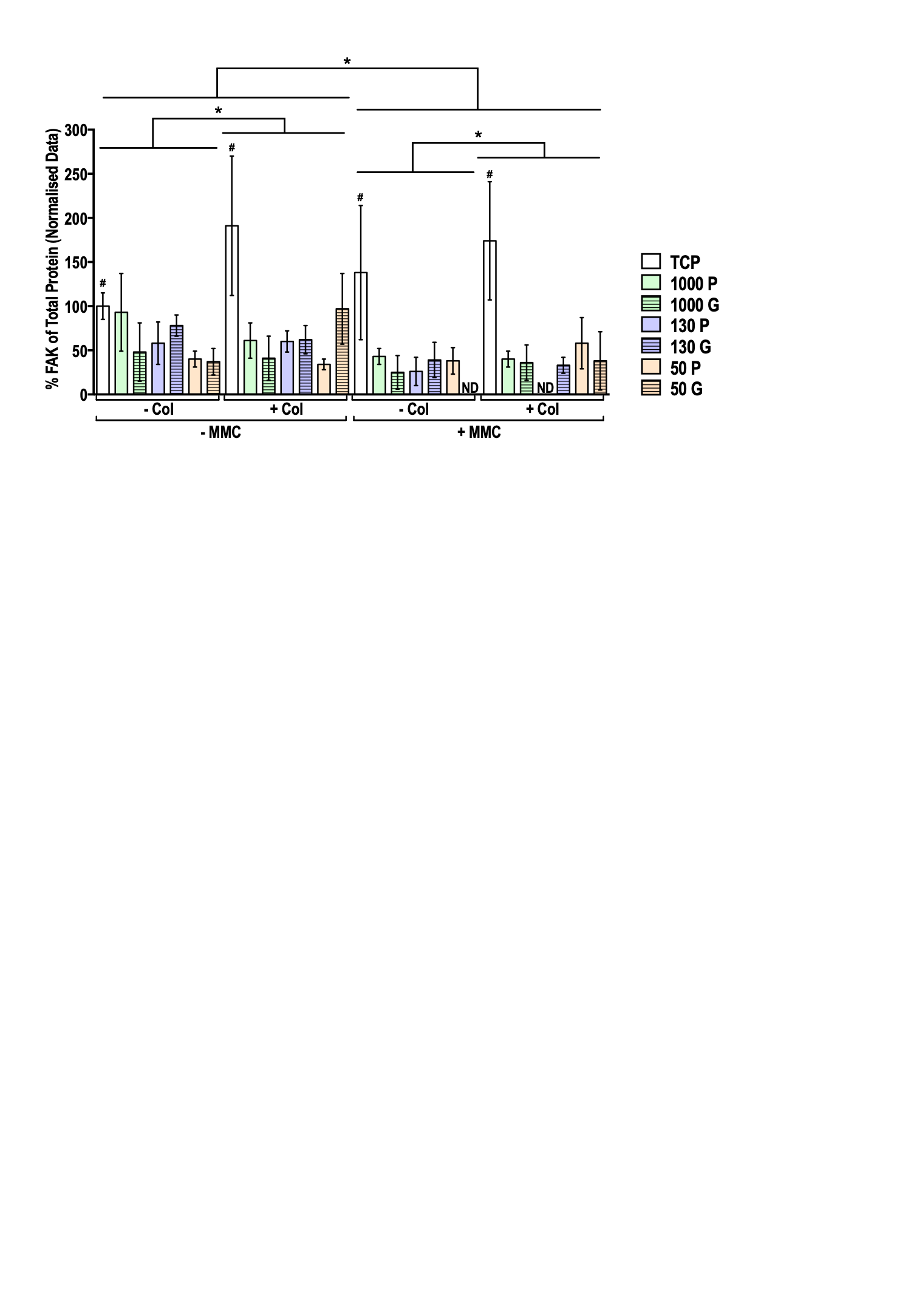


**Figure S10:** Human dermal fibroblast number on tissue culture plastic (TCP) without and with collagen type I coating (- Col, + Col) and macromolecular crowding (- MMC, + MMC) and on substrates of varying stiffness (1,000 kPa, 130 kPa, 50 kPa), surface topography [planar (P), grooved (G)], collagen type I coating (- Col, + Col) and macromolecular crowding (- MMC, + MMC) at day 3, day 7 and day 14. * indicates statistically significant difference (*p* < 0.05) between without and with collagen type I coating and between without and with MMC and # indicates statistical difference (*p* < 0.05) between TCP and PDMS substrates.


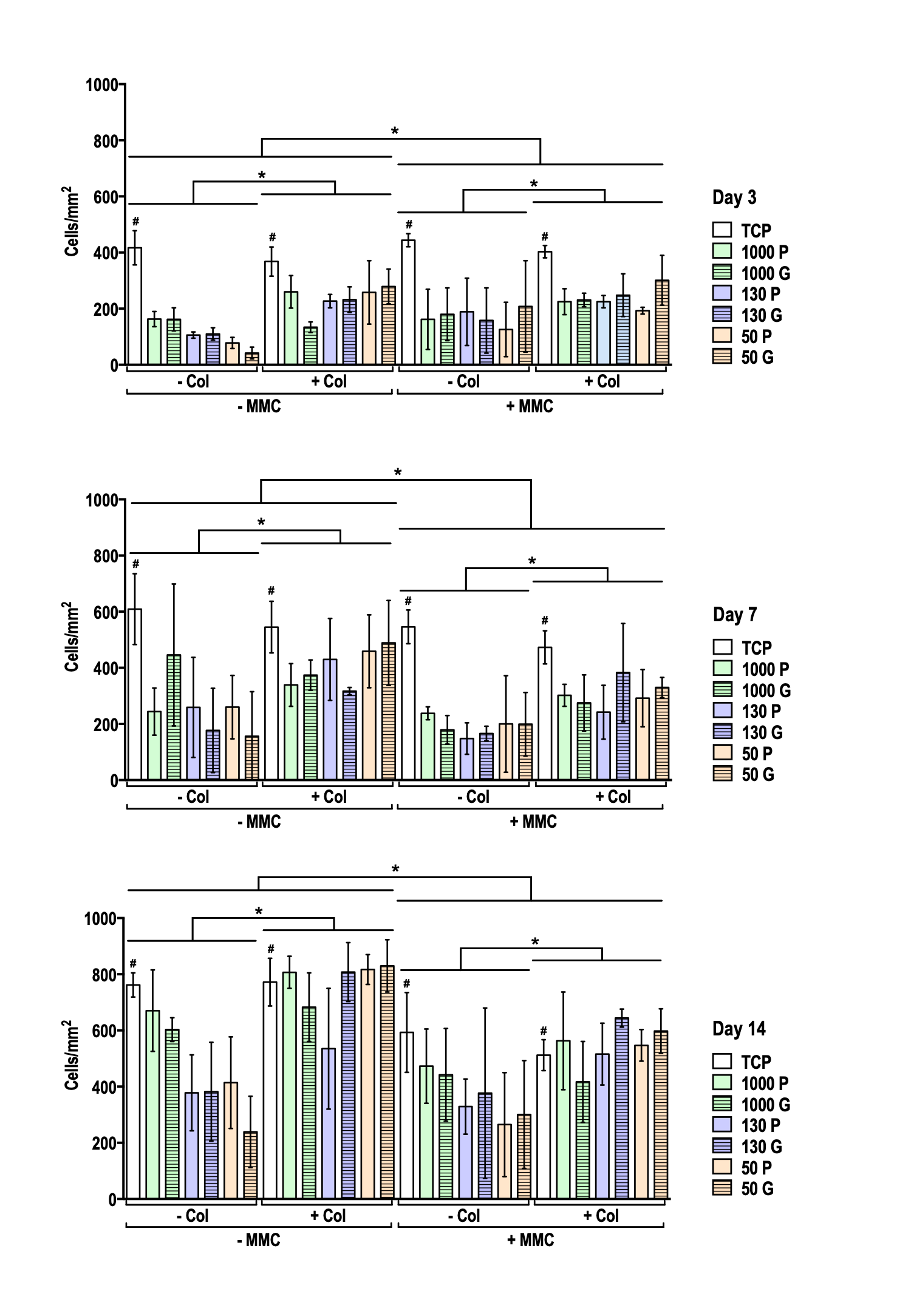


**Figure S11:** Human dermal fibroblast metabolic activity on tissue culture plastic (TCP) without and with collagen type I coating (- Col, + Col) and macromolecular crowding (- MMC, + MMC) and on substrates of varying stiffness (1,000 kPa, 130 kPa, 50 kPa), surface topography [planar (P), grooved (G)], collagen type I coating (- Col, + Col) and macromolecular crowding (- MMC, + MMC) at day 3, day 7 and day 14. * indicates statistically significant difference (*p* < 0.05) between without and with collagen type I coating and between without and with MMC, # indicates statistical difference (*p* < 0.05) between TCP and PDMS substrates and + indicates statistical difference (*p* < 0.05) between planar and grooved topography.


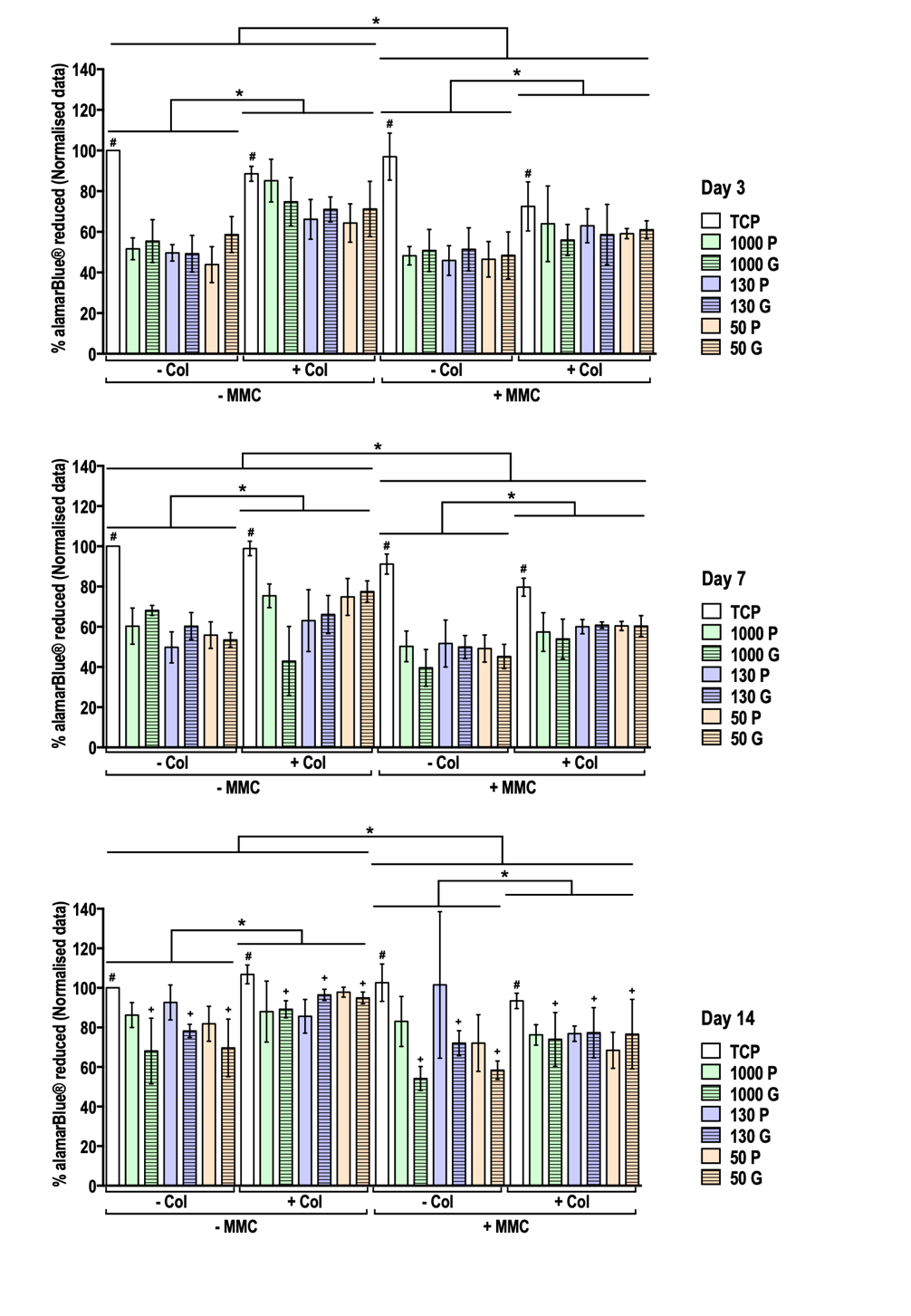


**Figure S12:** Human dermal fibroblast viability on tissue culture plastic (TCP) without and with collagen type I coating (- Col, + Col) and macromolecular crowding (- MMC, + MMC) and on substrates of varying stiffness (1,000 kPa, 130 kPa, 50 kPa), surface topography (planar, grooved), collagen type I coating (- Col, + Col) and macromolecular crowding (- MMC, + MMC) at day 3, day 7 and day 14, as shown using Live/Dead® assay. Live cells are represented in green and dead cells are represented in red. Cell viability was estimated at > 95 % for all conditions. Scale bar = 50 *μ*m.


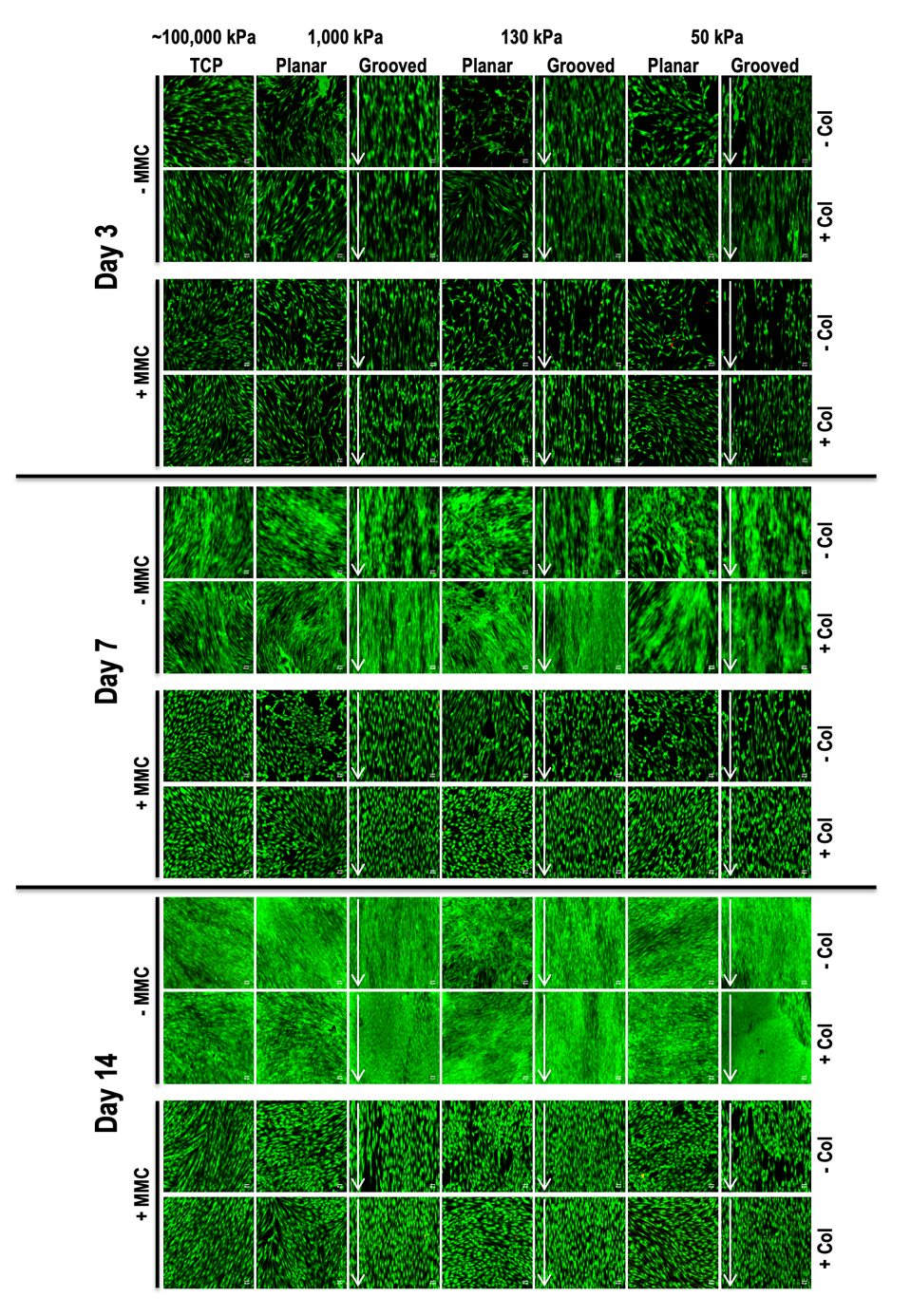


**Figure S13:** Human dermal fibroblast deposited collagen type I matrix and quantification of collagen type I matrix area deposited per cell at day 3 on tissue culture plastic (TCP) without and with collagen type I coating (- Col, + Col) and macromolecular crowding (- MMC, + MMC) and on substrates of varying stiffness (1,000 kPa, 130 kPa, 50 kPa), surface topography [planar (P), grooved (G)], collagen type I coating (- Col, + Col) and macromolecular crowding (- MMC, + MMC). Collagen type I is represented in orange. DAPI is represented in blue. Scale bar = 50 *µ*m. * indicates statistically significant difference (*p* < 0.05) between without and with MMC.


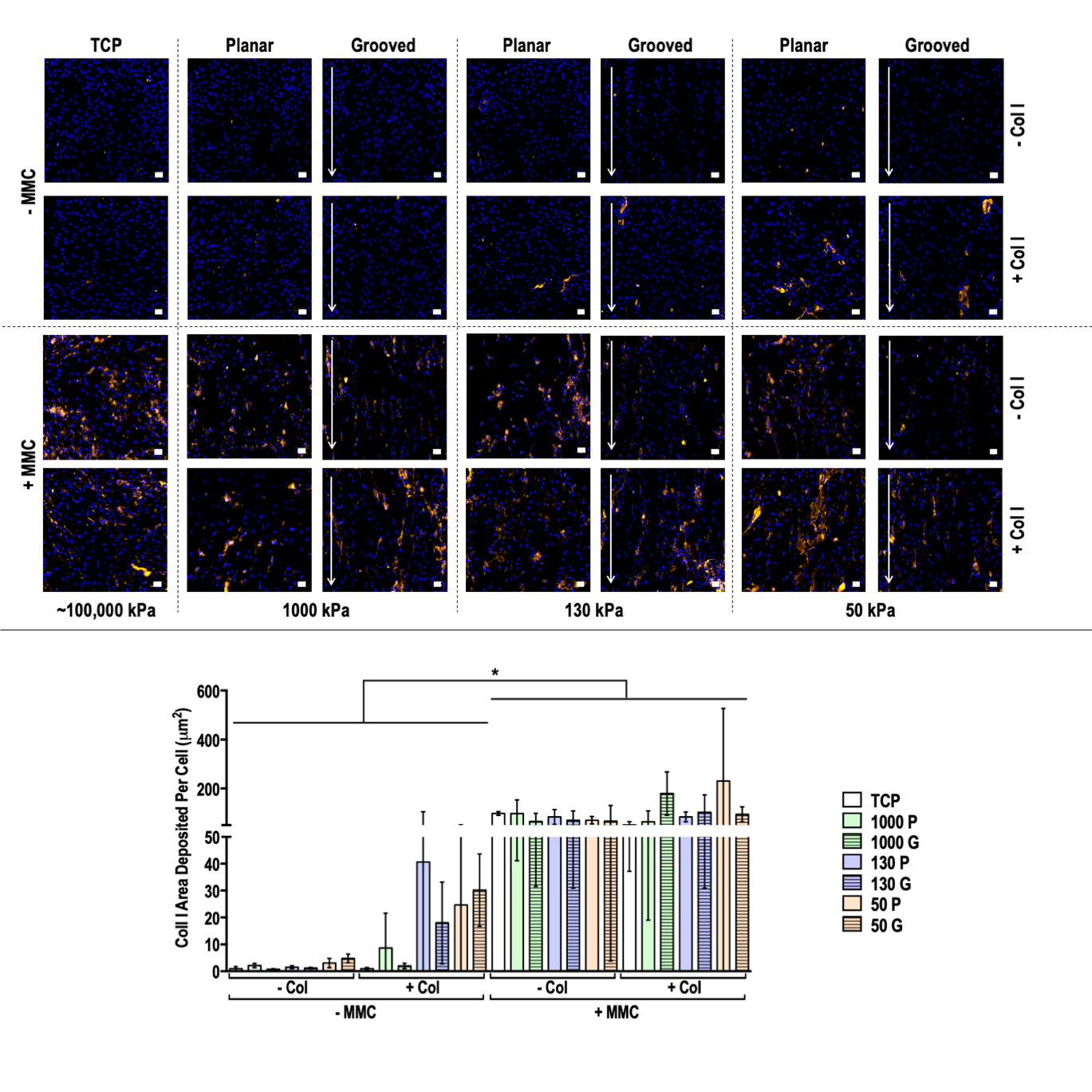


**Figure S14:** Human dermal fibroblast deposited collagen type III matrix and quantification of collagen type III matrix area deposited per cell at day 3 on tissue culture plastic (TCP) without and with collagen type I coating (- Col, + Col) and macromolecular crowding (- MMC, + MMC) and on substrates of varying stiffness (1,000 kPa, 130 kPa, 50 kPa), surface topography [planar (P), grooved (G)], collagen type I coating (- Col, + Col) and macromolecular crowding (- MMC, + MMC). Collagen type III is represented in green. DAPI is represented in blue. Scale bar = 50 *µ*m. * indicates statistically significant difference (*p* < 0.05) between without and with MMC and + indicates statistical difference (*p* < 0.05) between planar and grooved topography.


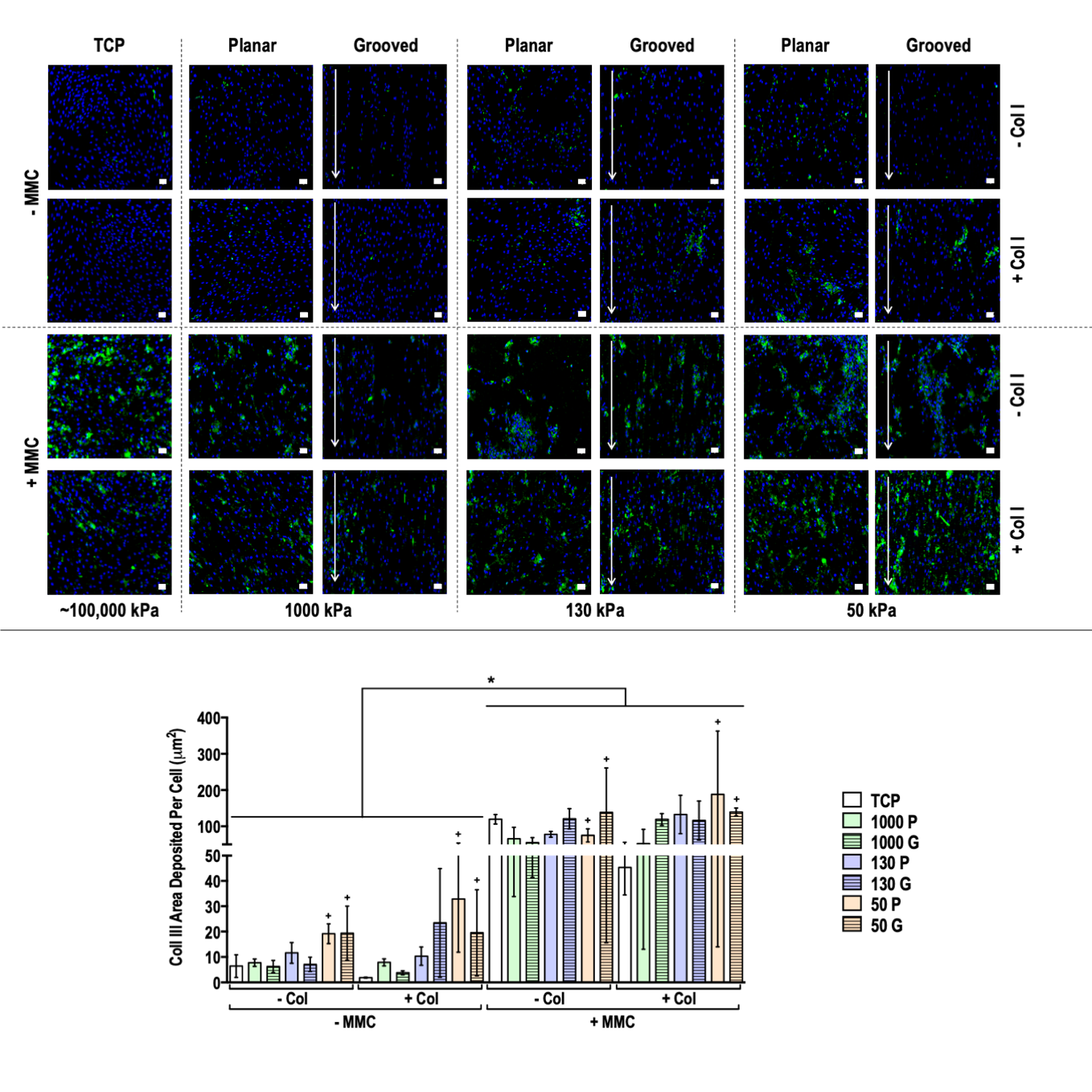


**Figure S15:** Human dermal fibroblast deposited collagen type IV matrix and quantification of collagen type IV matrix area deposited per cell at day 3 on tissue culture plastic (TCP) without and with collagen type I coating (- Col, + Col) and macromolecular crowding (- MMC, + MMC) and on substrates of varying stiffness (1,000 kPa, 130 kPa, 50 kPa), surface topography [planar (P), grooved (G)], collagen type I coating (- Col, + Col) and macromolecular crowding (- MMC, + MMC). Collagen type IV is represented in red. DAPI is represented in blue. Scale bar = 50 *µ*m. * indicates statistically significant difference (*p* < 0.05) between without and with collagen type I coating and between without and with MMC, # indicates statistical difference (*p* < 0.05) between TCP and PDMS substrates and + indicates statistical difference (*p* < 0.05) between planar and grooved topography.


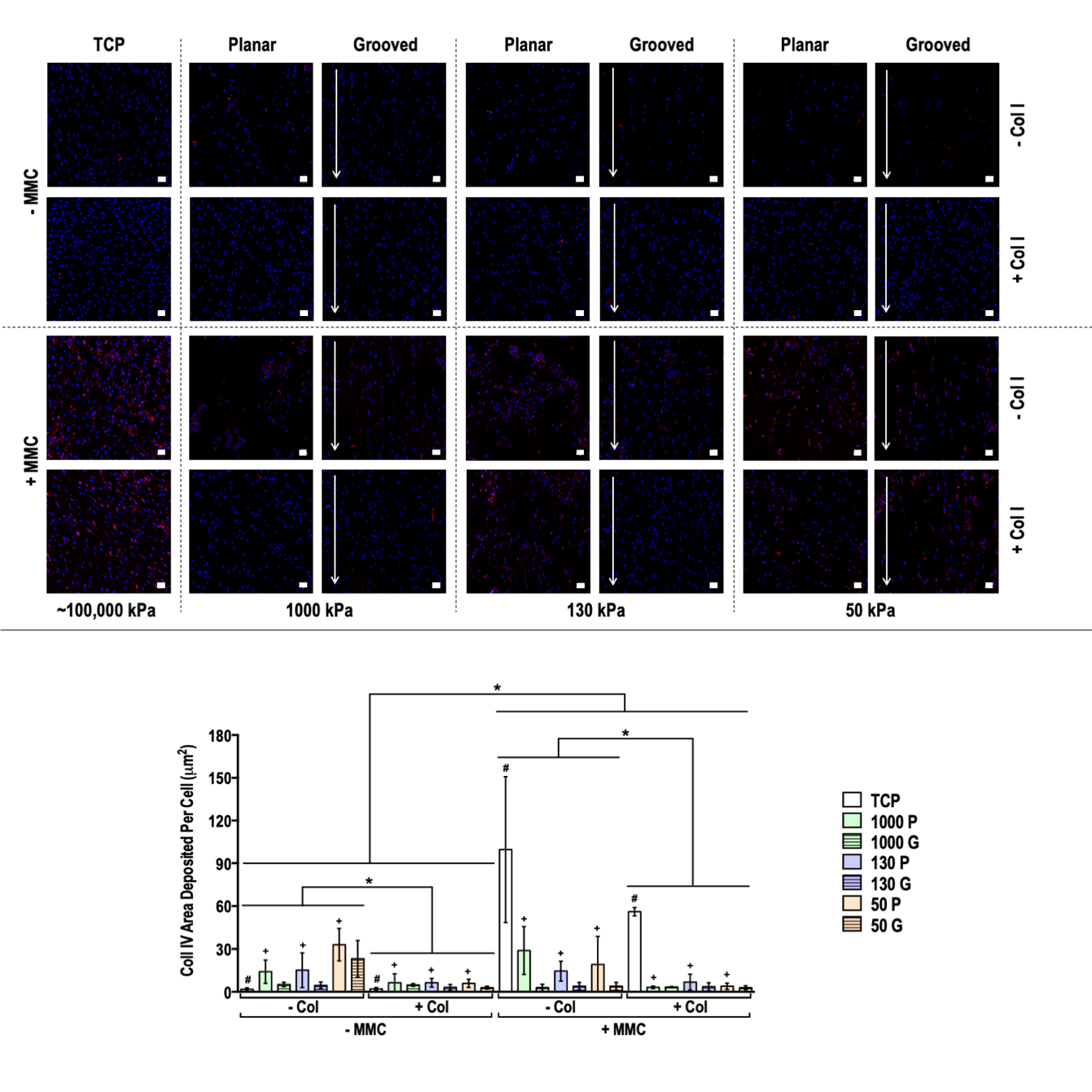


**Figure S16:** Human dermal fibroblast deposited collagen type V matrix and quantification of collagen type V matrix area deposited per cell at day 3 on tissue culture plastic (TCP) without and with collagen type I coating (- Col, + Col) and macromolecular crowding (- MMC, + MMC) and on substrates of varying stiffness (1,000 kPa, 130 kPa, 50 kPa), surface topography [planar (P), grooved (G)], collagen type I coating (- Col, + Col) and macromolecular crowding (- MMC, + MMC). Collagen type V is represented in green. DAPI is represented in blue. Scale bar = 50 *µ*m. * indicates statistically significant difference (*p* < 0.05) between without and with MMC.


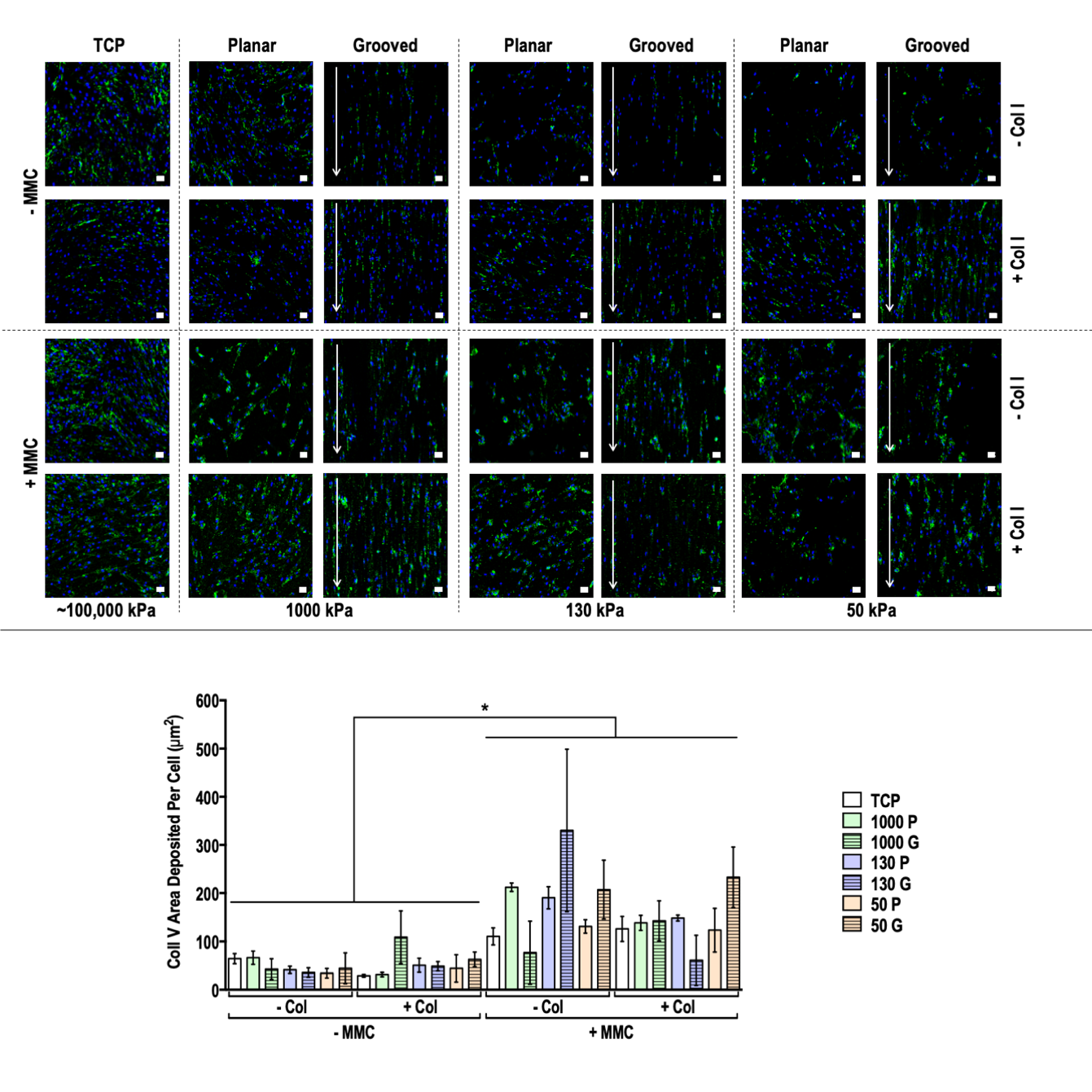


**Figure S17:** Human dermal fibroblast deposited collagen type VI matrix and quantification of collagen type VI matrix area deposited per cell at day 3 on tissue culture plastic (TCP) without and with collagen type I coating (- Col, + Col) and macromolecular crowding (- MMC, + MMC) and on substrates of varying stiffness (1,000 kPa, 130 kPa, 50 kPa), surface topography [planar (P), grooved (G)], collagen type I coating (- Col, + Col) and macromolecular crowding (- MMC, + MMC). Collagen type VI is represented in red. DAPI is represented in blue. Scale bar = 50 *µ*m. * indicates statistically significant difference (*p* < 0.05) between without and with collagen type I coating and between without and with MMC and + indicates statistical difference (*p* < 0.05) between planar and grooved topography.


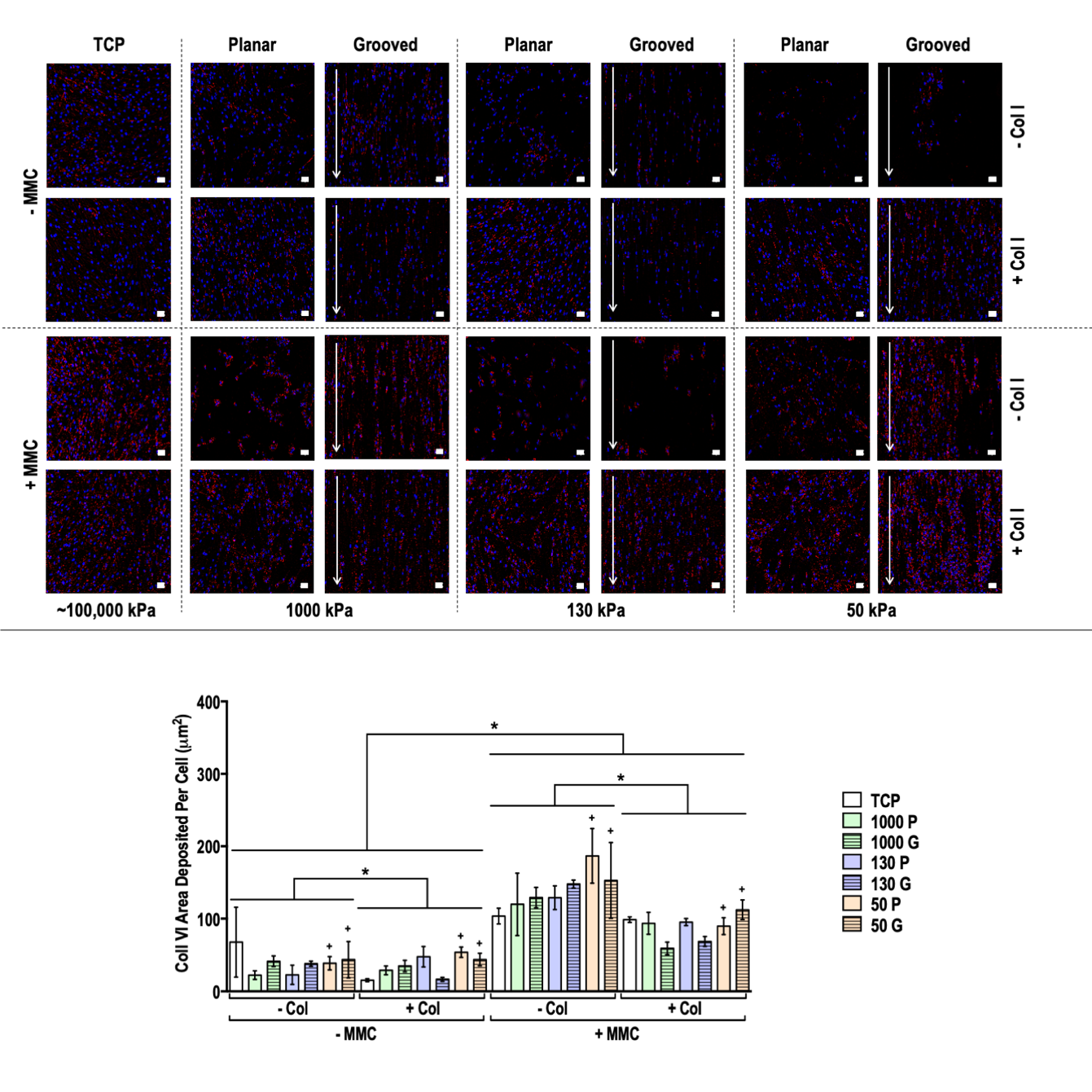


**Figure S18:** Human dermal fibroblast deposited fibronectin matrix and quantification of fibronectin matrix area deposited per cell at day 3 on tissue culture plastic (TCP) without and with collagen type I coating (- Col, + Col) and macromolecular crowding (- MMC, + MMC) and on substrates of varying stiffness (1,000 kPa, 130 kPa, 50 kPa), surface topography [planar (P), grooved (G)], collagen type I coating (- Col, + Col) and macromolecular crowding (- MMC, + MMC). Fibronectin is represented in green. DAPI is represented in blue. Scale bar = 50 *µ*m. * indicates statistically significant difference (*p* < 0.05) between without and with MMC.


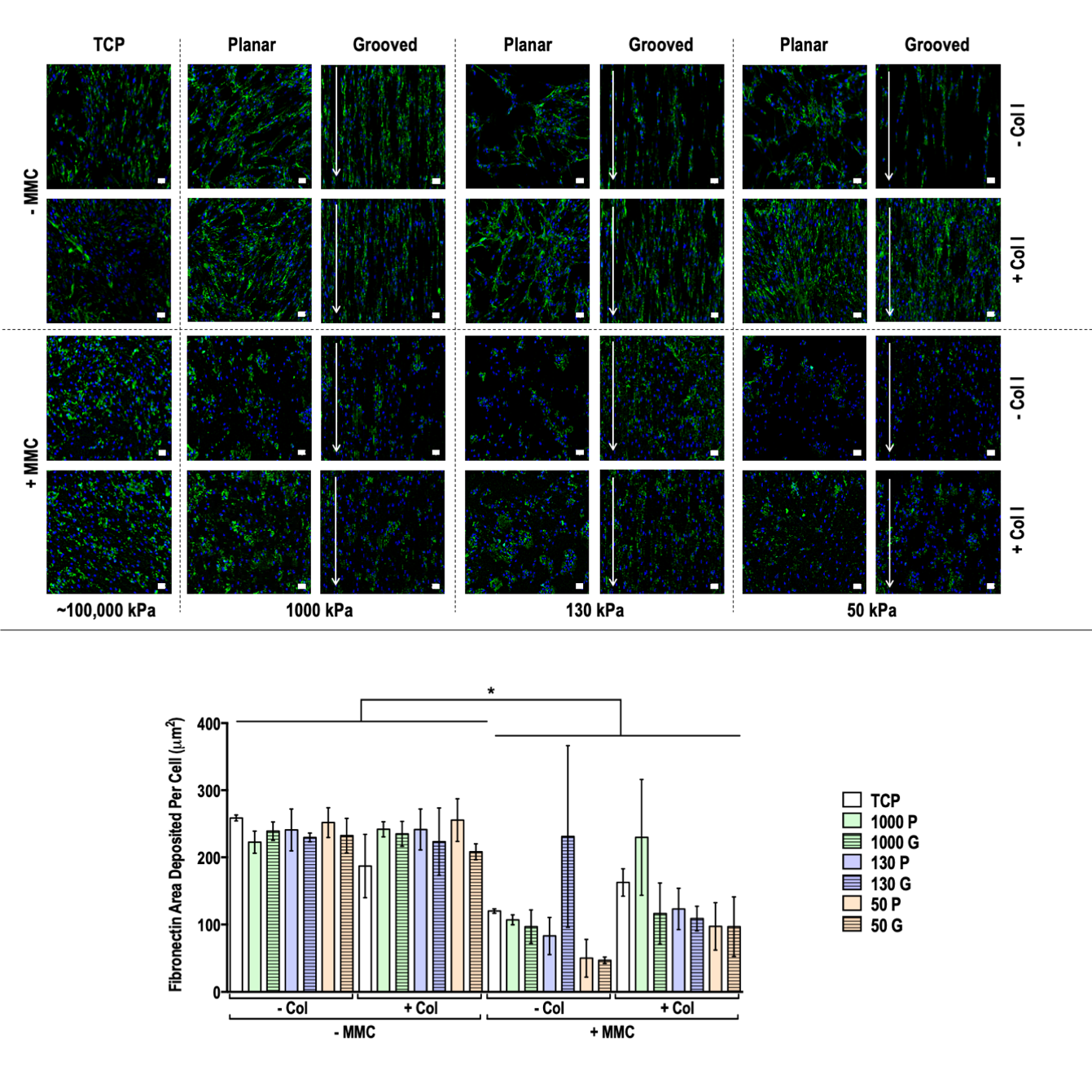


**Figure S19:** Human dermal fibroblast deposited collagen type I matrix and quantification of collagen type I matrix area deposited per cell at day 7 on tissue culture plastic (TCP) without and with collagen type I coating (- Col, + Col) and macromolecular crowding (- MMC, + MMC) and on substrates of varying stiffness (1,000 kPa, 130 kPa, 50 kPa), surface topography [planar (P), grooved (G)], collagen type I coating (- Col, + Col) and macromolecular crowding (- MMC, + MMC). Collagen type I is represented in orange. DAPI is represented in blue. Scale bar = 50 *µ*m. * indicates statistically significant difference (*p* < 0.05) between without and with collagen type I coating and between without and with MMC, # indicates statistical difference (*p* < 0.05) between TCP and PDMS substrates and + indicates statistical difference (*p* < 0.05) between planar and grooved topography.


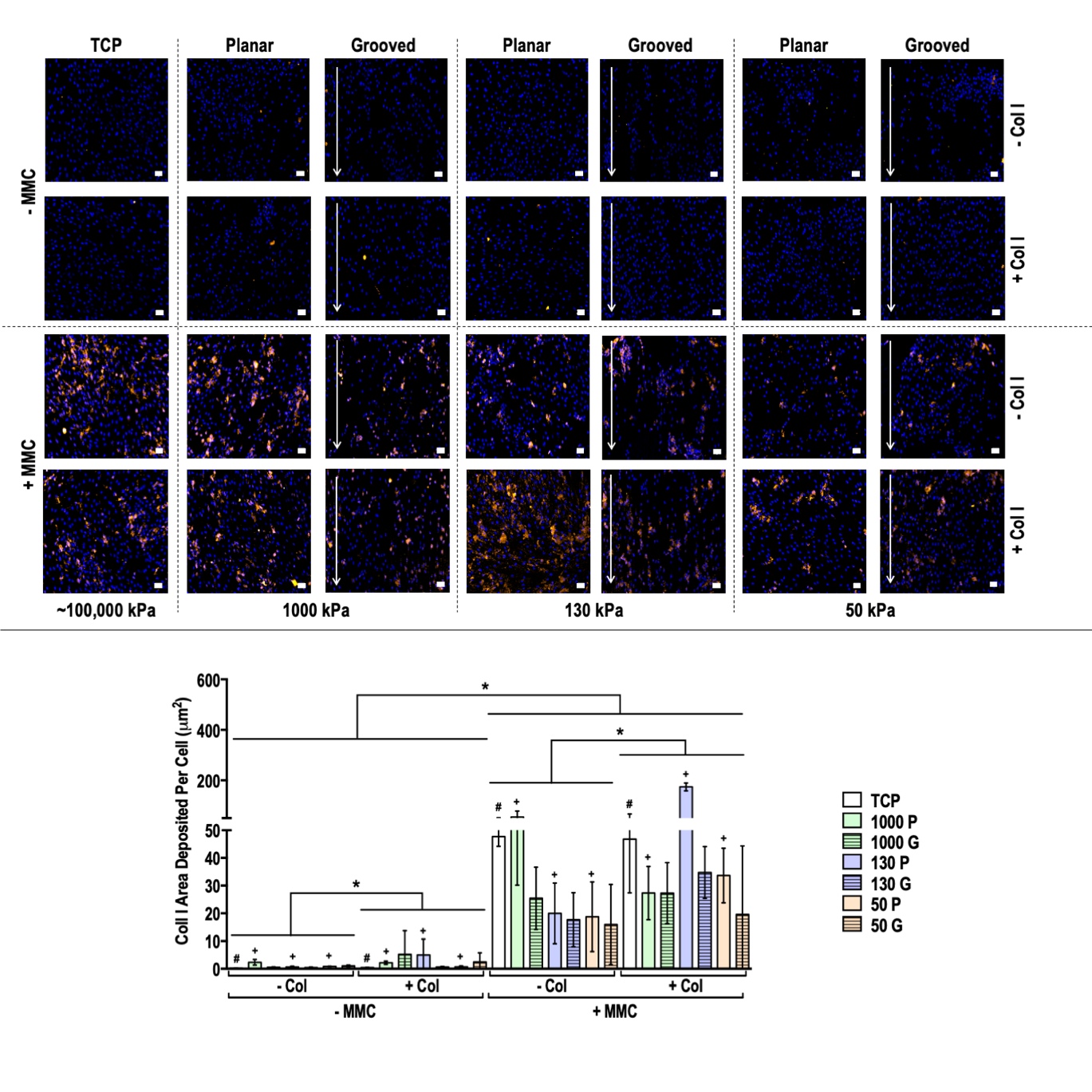


**Figure S20:** Human dermal fibroblast deposited collagen type III matrix and quantification of collagen type III matrix area deposited per cell at day 7 on tissue culture plastic (TCP) without and with collagen type I coating (- Col, + Col) and macromolecular crowding (- MMC, + MMC) and on substrates of varying stiffness (1,000 kPa, 130 kPa, 50 kPa), surface topography [planar (P), grooved (G)], collagen type I coating (- Col, + Col) and macromolecular crowding (- MMC, + MMC). Collagen type III is represented in green. DAPI is represented in blue. Scale bar = 50 *µ*m. * indicates statistically significant difference (*p* < 0.05) between without and with collagen type I coating and between without and with MMC.


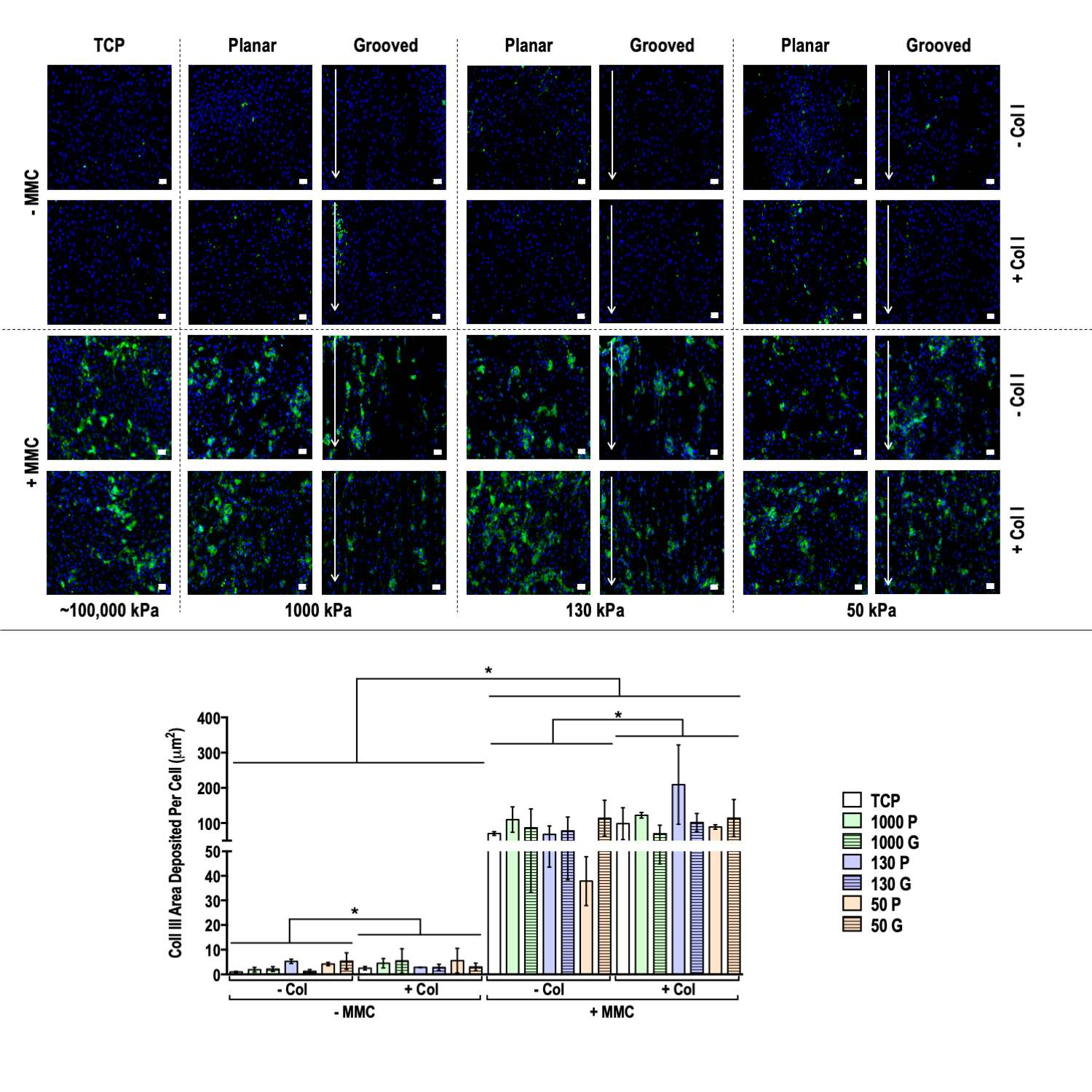


**Figure S21:** Human dermal fibroblast deposited collagen type IV matrix and quantification of collagen type IV matrix area deposited per cell at day 7 on tissue culture plastic (TCP) without and with collagen type I coating (- Col, + Col) and macromolecular crowding (- MMC, + MMC) and on substrates of varying stiffness (1,000 kPa, 130 kPa, 50 kPa), surface topography [planar (P), grooved (G)], collagen type I coating (- Col, + Col) and macromolecular crowding (- MMC, + MMC). Collagen type IV is represented in red. DAPI is represented in blue. Scale bar = 50 *µ*m. * indicates statistically significant difference (*p* < 0.05) between without and with MMC and # indicates statistical difference (*p* < 0.05) between TCP and PDMS substrates.


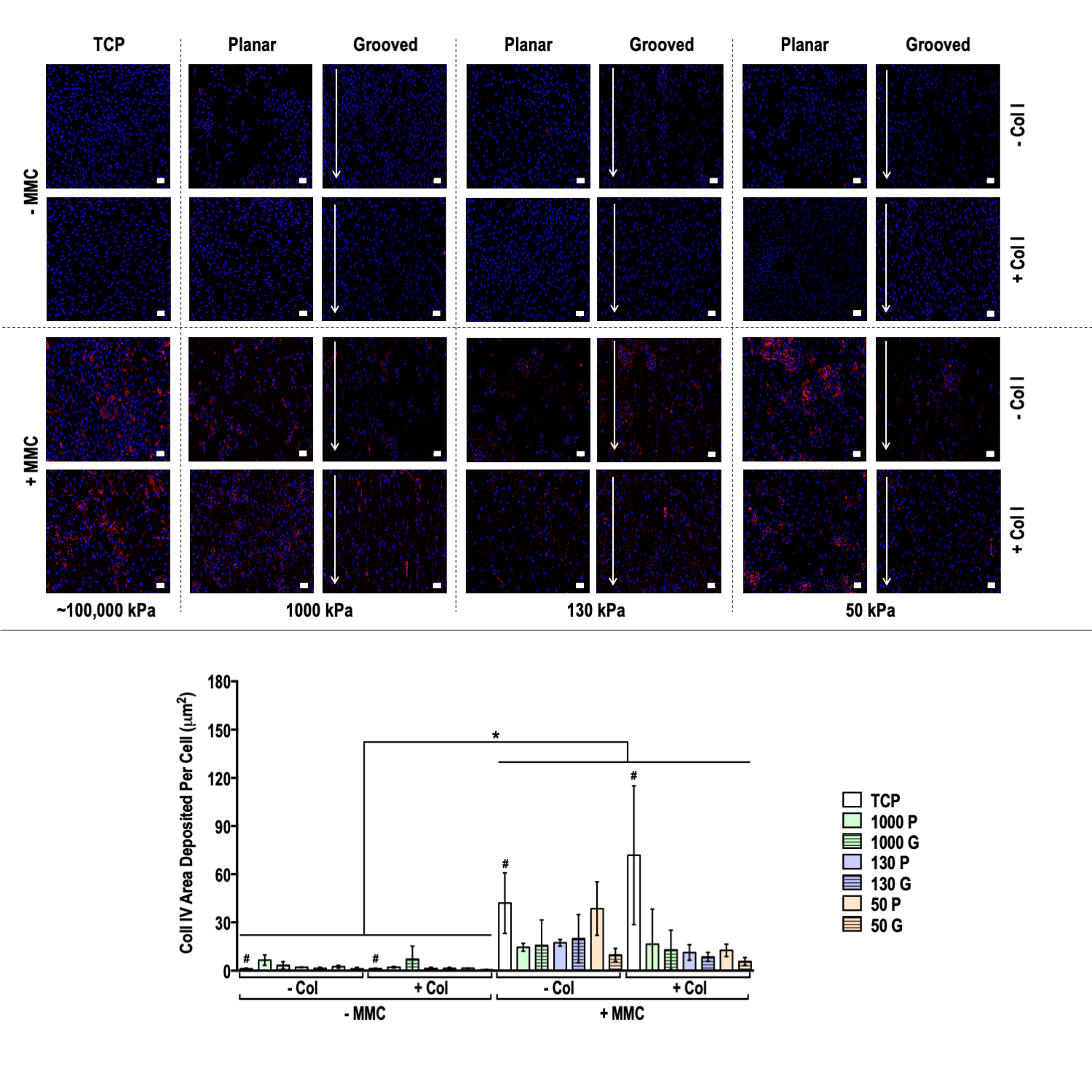


**Figure S22:** Human dermal fibroblast deposited collagen type V matrix and quantification of collagen type V matrix area deposited per cell at day 7 on tissue culture plastic (TCP) without and with collagen type I coating (- Col, + Col) and macromolecular crowding (- MMC, + MMC) and on substrates of varying stiffness (1,000 kPa, 130 kPa, 50 kPa), surface topography [planar (P), grooved (G)], collagen type I coating (- Col, + Col) and macromolecular crowding (- MMC, + MMC). Collagen type V is represented in green. DAPI is represented in blue. Scale bar = 50 *µ*m. * indicates statistically significant difference (*p* < 0.05) between without and with collagen type I coating and between without and with MMC, # indicates statistical difference (*p* < 0.05) between TCP and PDMS substrates and + indicates statistical difference (*p* < 0.05) between planar and grooved topography.


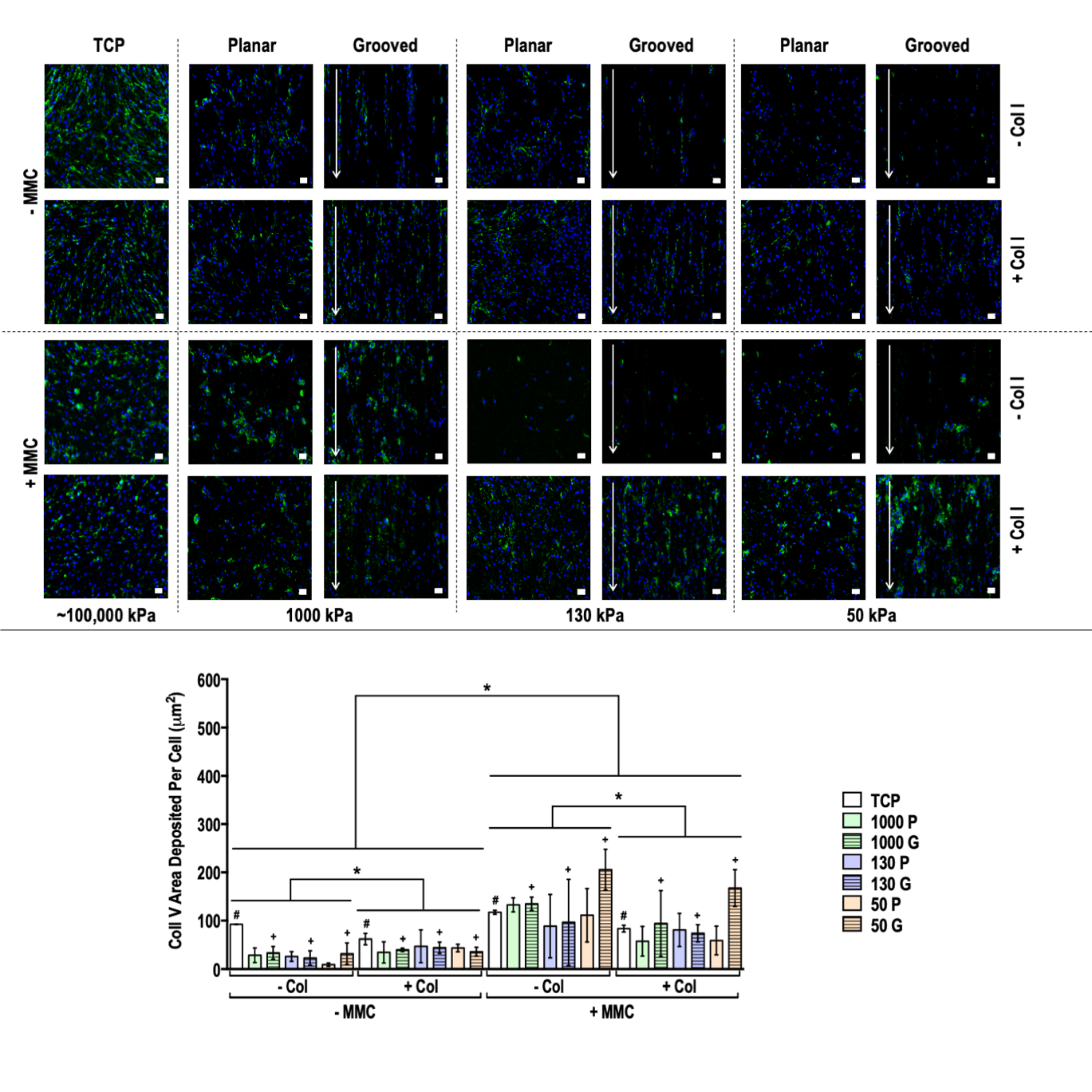


**Figure S23:** Human dermal fibroblast deposited collagen type VI matrix and quantification of collagen type VI matrix area deposited per cell at day 7 on tissue culture plastic (TCP) without and with collagen type I coating (- Col, + Col) and macromolecular crowding (- MMC, + MMC) and on substrates of varying stiffness (1,000 kPa, 130 kPa, 50 kPa), surface topography [planar (P), grooved (G)], collagen type I coating (- Col, + Col) and macromolecular crowding (- MMC, + MMC). Collagen type VI is represented in red. DAPI is represented in blue. Scale bar = 50 *µ*m. * indicates statistically significant difference (*p* < 0.05) between without and with MMC.


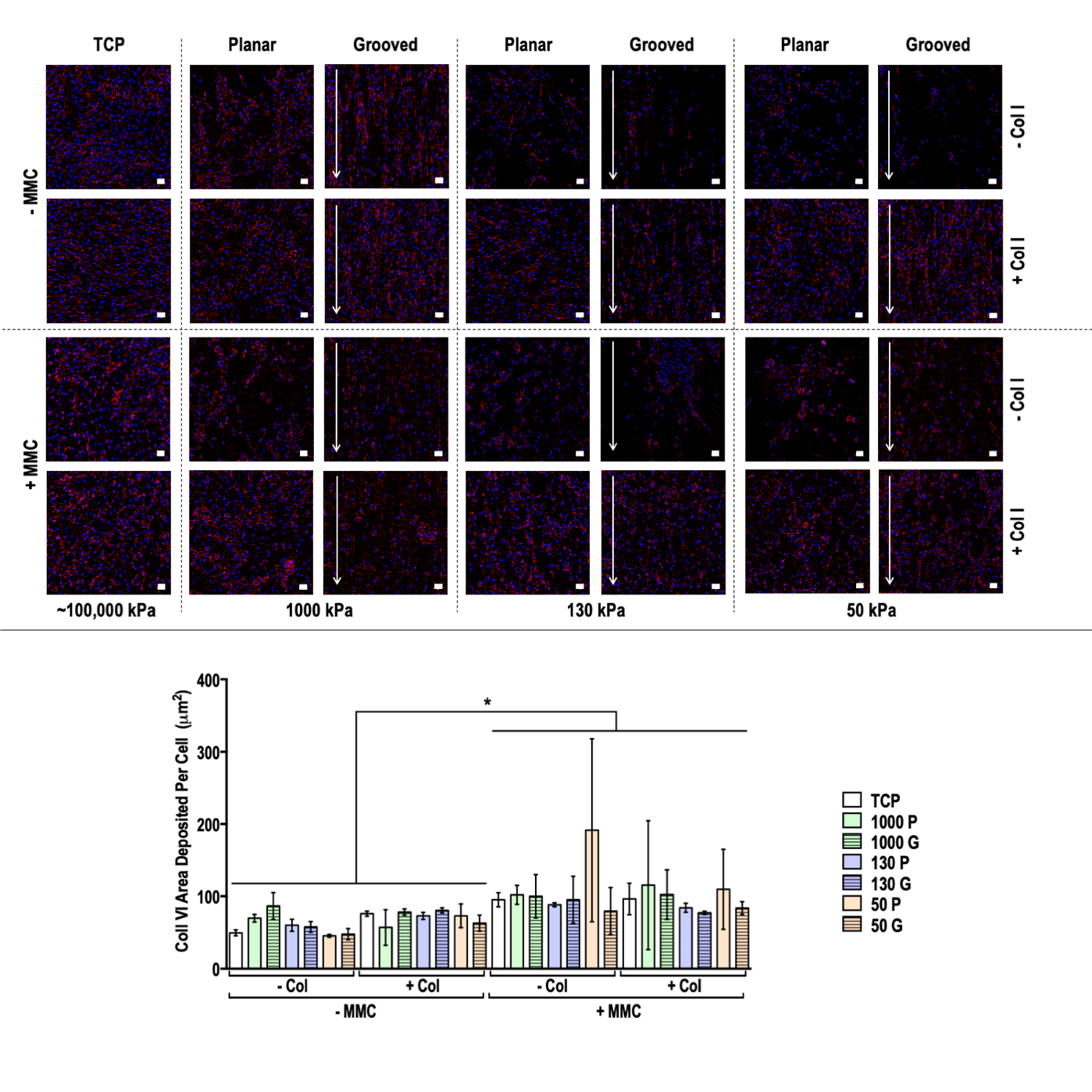


**Figure S24:** Human dermal fibroblast deposited fibronectin matrix and quantification of fibronectin matrix area deposited per cell at day 7 on tissue culture plastic (TCP) without and with collagen type I coating (- Col, + Col) and macromolecular crowding (- MMC, + MMC) and on substrates of varying stiffness (1,000 kPa, 130 kPa, 50 kPa), surface topography [planar (P), grooved (G)], collagen type I coating (- Col, + Col) and macromolecular crowding (- MMC, + MMC). Fibronectin is represented in green. DAPI is represented in blue. Scale bar = 50 *µ*m. * indicates statistically significant difference (*p* < 0.05) between without and with MMC and # indicates statistical difference (*p* < 0.05) between TCP and PDMS substrates.


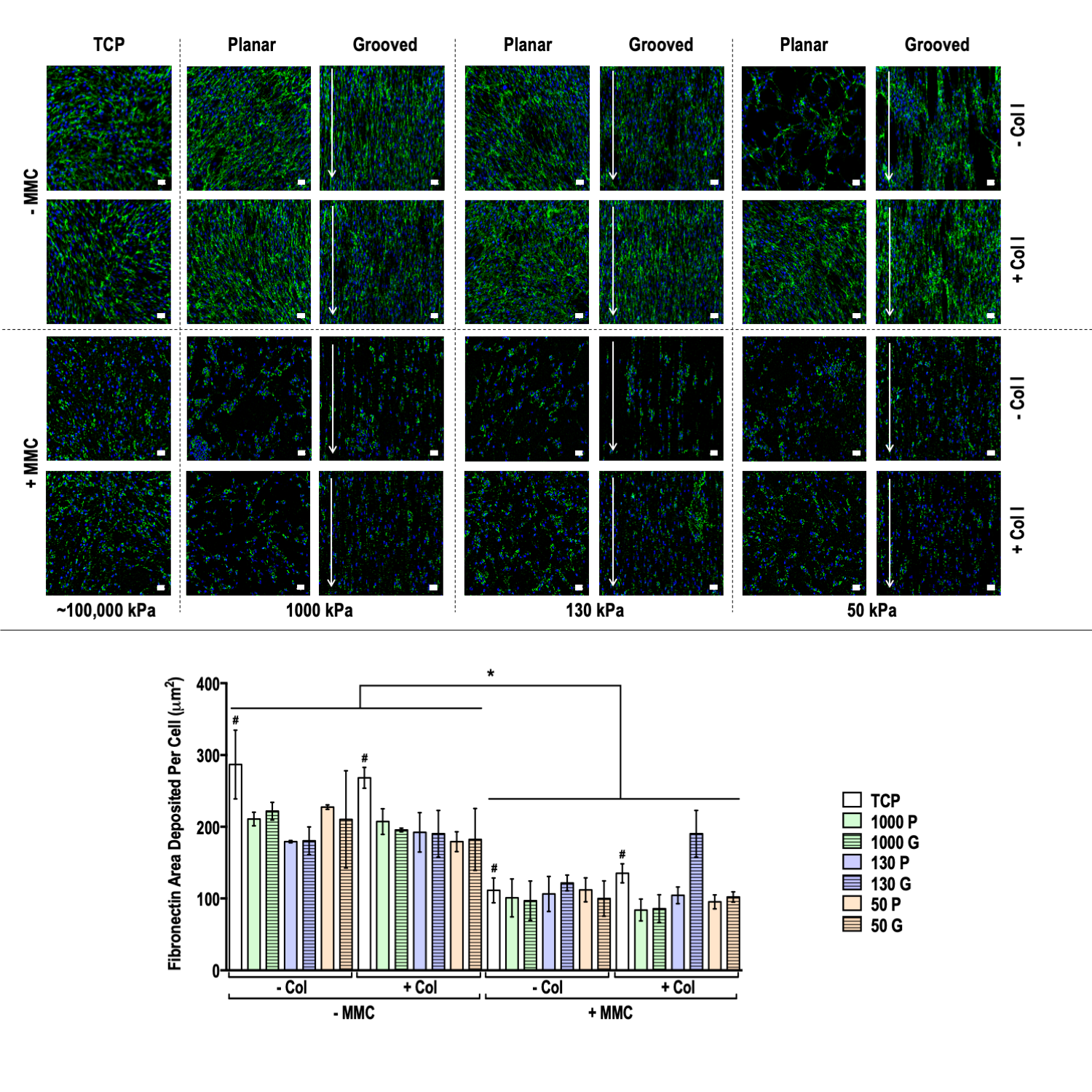


**Figure S25:** Human dermal fibroblast deposited collagen type V matrix and quantification of collagen type V matrix area deposited per cell at day 14 on tissue culture plastic (TCP) without and with collagen type I coating (- Col, + Col) and macromolecular crowding (- MMC, + MMC) and on substrates of varying stiffness (1,000 kPa, 130 kPa, 50 kPa), surface topography [planar (P), grooved (G)], collagen type I coating (- Col, + Col) and macromolecular crowding (- MMC, + MMC). Collagen type V is presented in green. DAPI is presented in blue. Scale bar = 50 *µ*m. * indicates statistically significant difference (*p* < 0.05) between without and with MMC.


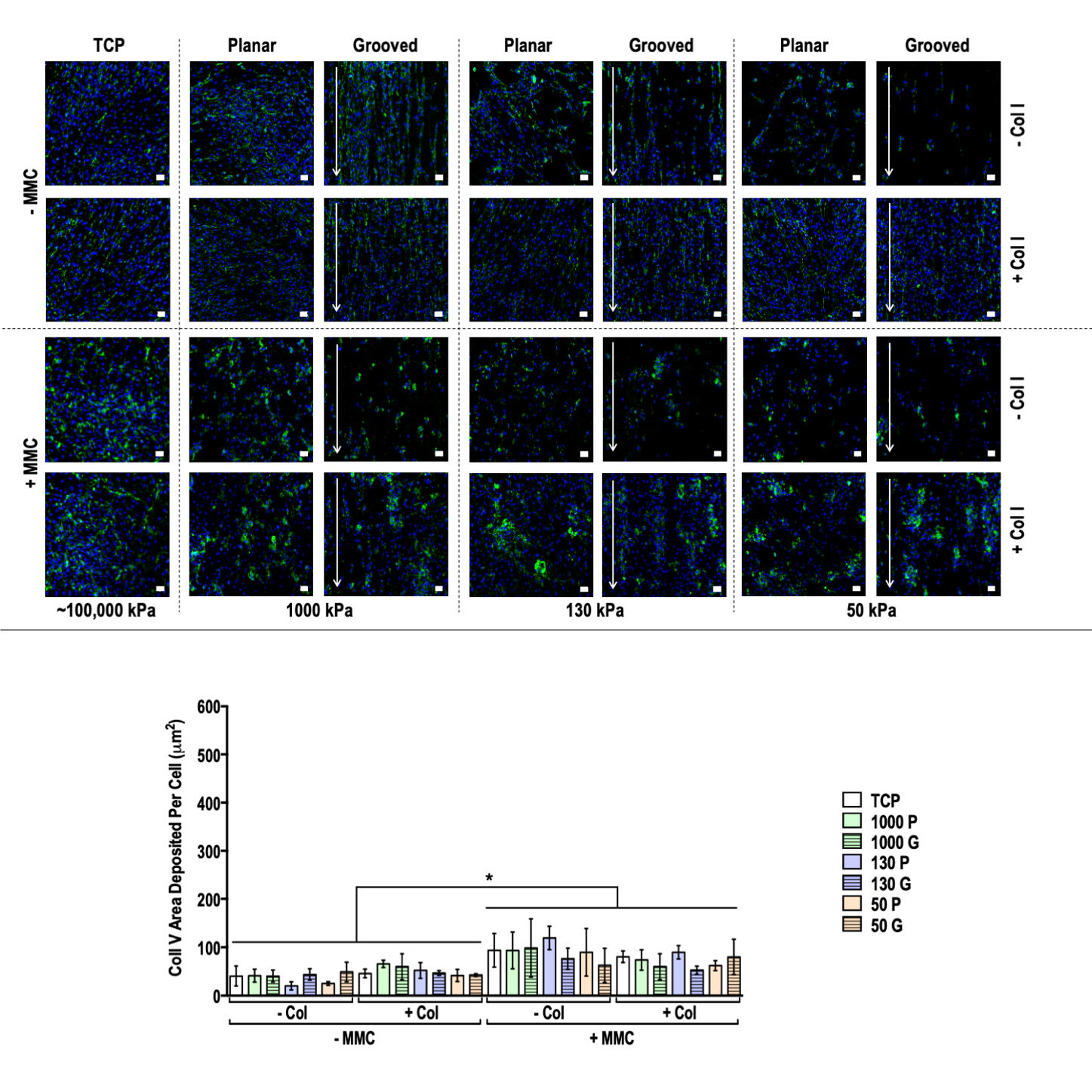


**Figure S26:** Human dermal fibroblast deposited collagen type VI matrix and quantification of collagen type VI matrix area deposited per cell at day 14 on tissue culture plastic (TCP) without and with collagen type I coating (- Col, + Col) and macromolecular crowding (- MMC, + MMC) and on substrates of varying stiffness (1,000 kPa, 130 kPa, 50 kPa), surface topography [planar (P), grooved (G)], collagen type I coating (- Col, + Col) and macromolecular crowding (- MMC, + MMC). Collagen type VI is represented in red. DAPI is represented in blue. Scale bar = 50 *µ*m. * indicates statistically significant difference (*p* < 0.05) between without and with collagen type I coating and between without and with MMC.


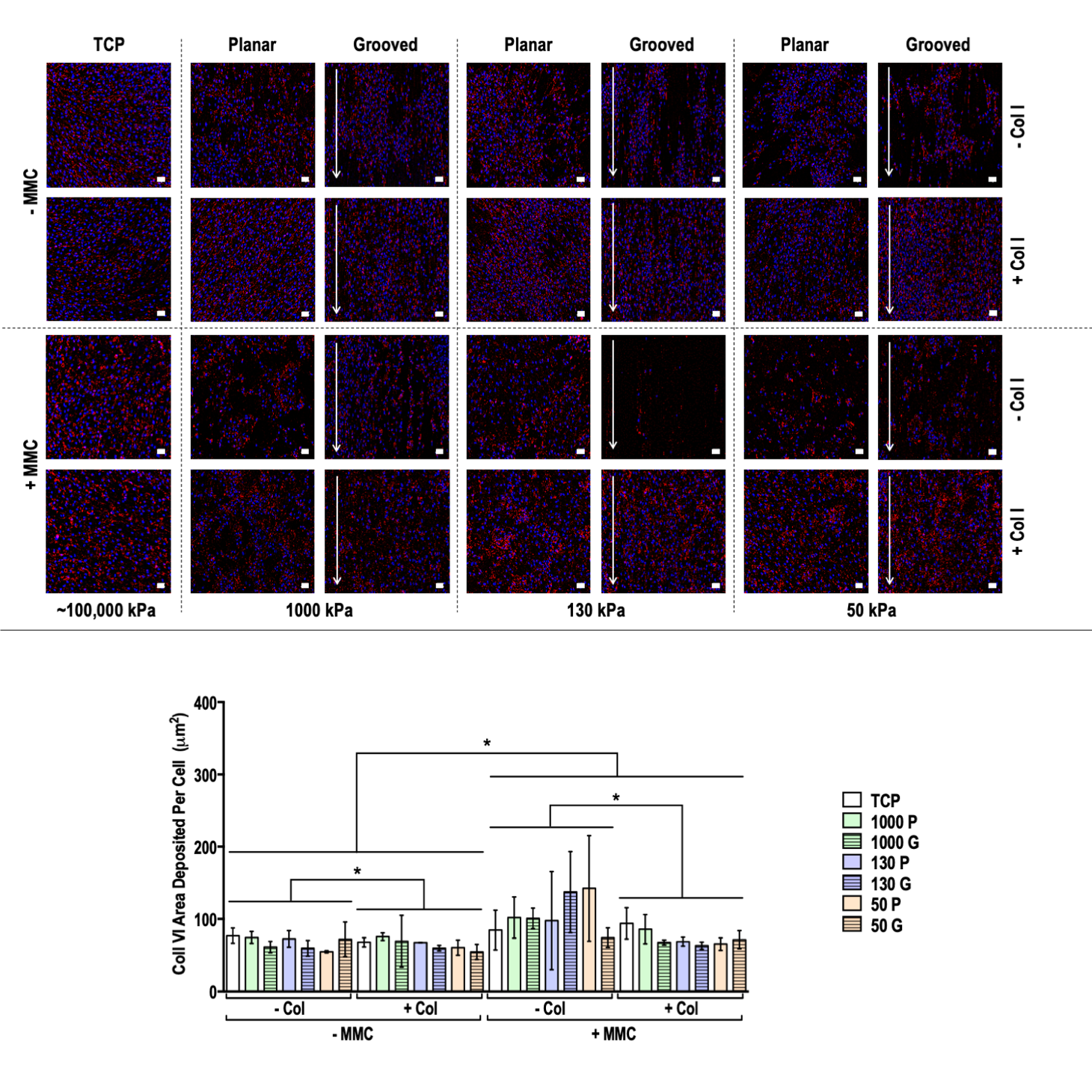


**Figure S27:** Human dermal fibroblast deposited fibronectin matrix and quantification of fibronectin matrix area deposited per cell at day 14 on tissue culture plastic (TCP) without and with collagen type I coating (- Col, + Col) and macromolecular crowding (- MMC, + MMC) and on substrates of varying stiffness (1,000 kPa, 130 kPa, 50 kPa), surface topography [planar (P), grooved (G)], collagen type I coating (- Col, + Col) and macromolecular crowding (- MMC, + MMC). Fibronectin is represented in green. DAPI is represented in blue. Scale bar = 50 *µ*m. * indicates statistically significant difference (*p* < 0.05) between without and with MMC and # indicates statistical difference (*p* < 0.05) between TCP and PDMS substrates.


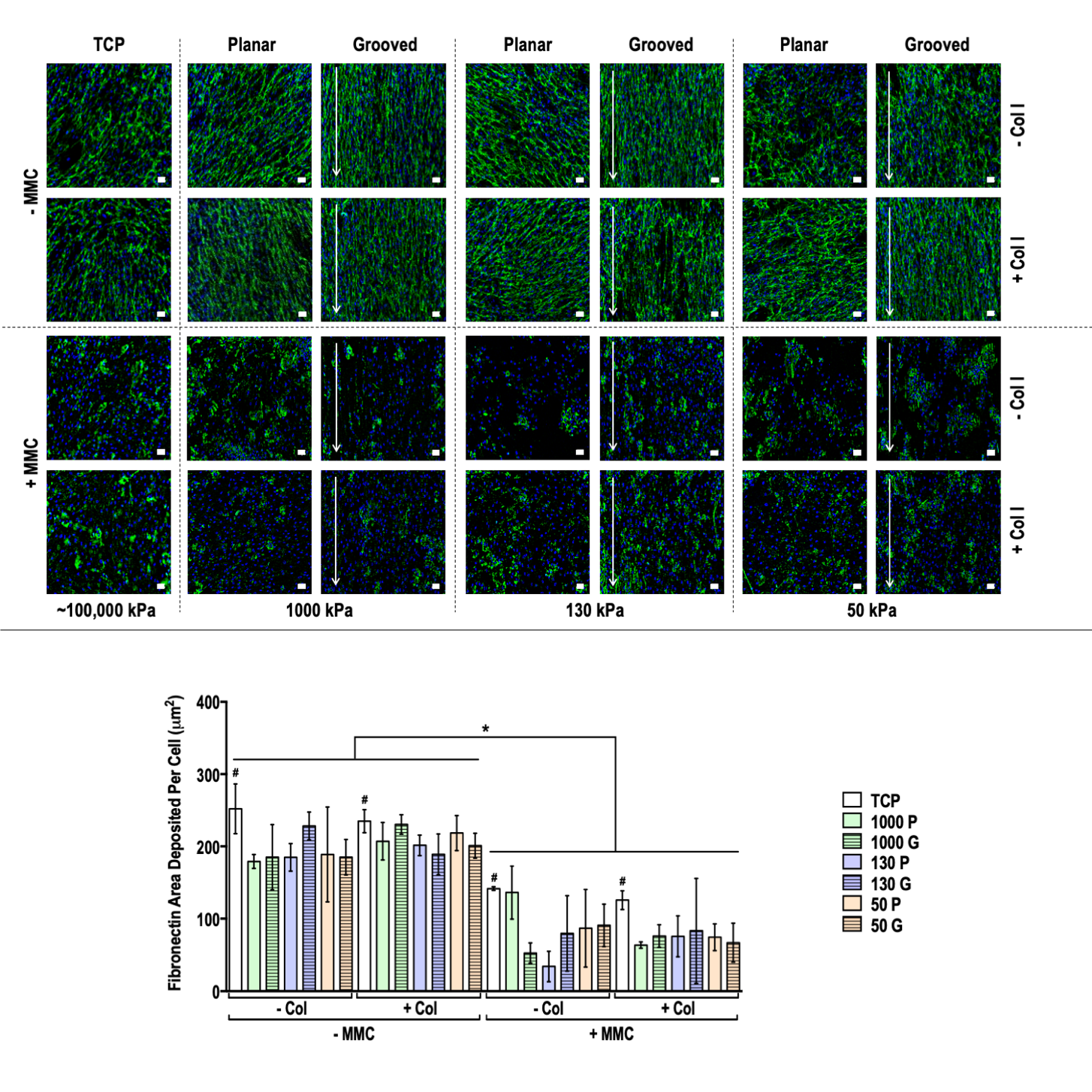


**Figure S28:** Human dermal fibroblast collagen type I matrix orientation at day 3, day 7 and day 14 on tissue culture plastic (TCP) without and with collagen type I coating (- Col, + Col) and macromolecular crowding (- MMC, + MMC) and on substrates of varying stiffness (1,000 kPa, 130 kPa, 50 kPa), surface topography [planar (P), grooved (G)], collagen type I coating (- Col, + Col) and macromolecular crowding (- MMC, + MMC). ND indicates conditions that did not produce sufficient matrix for analysis.


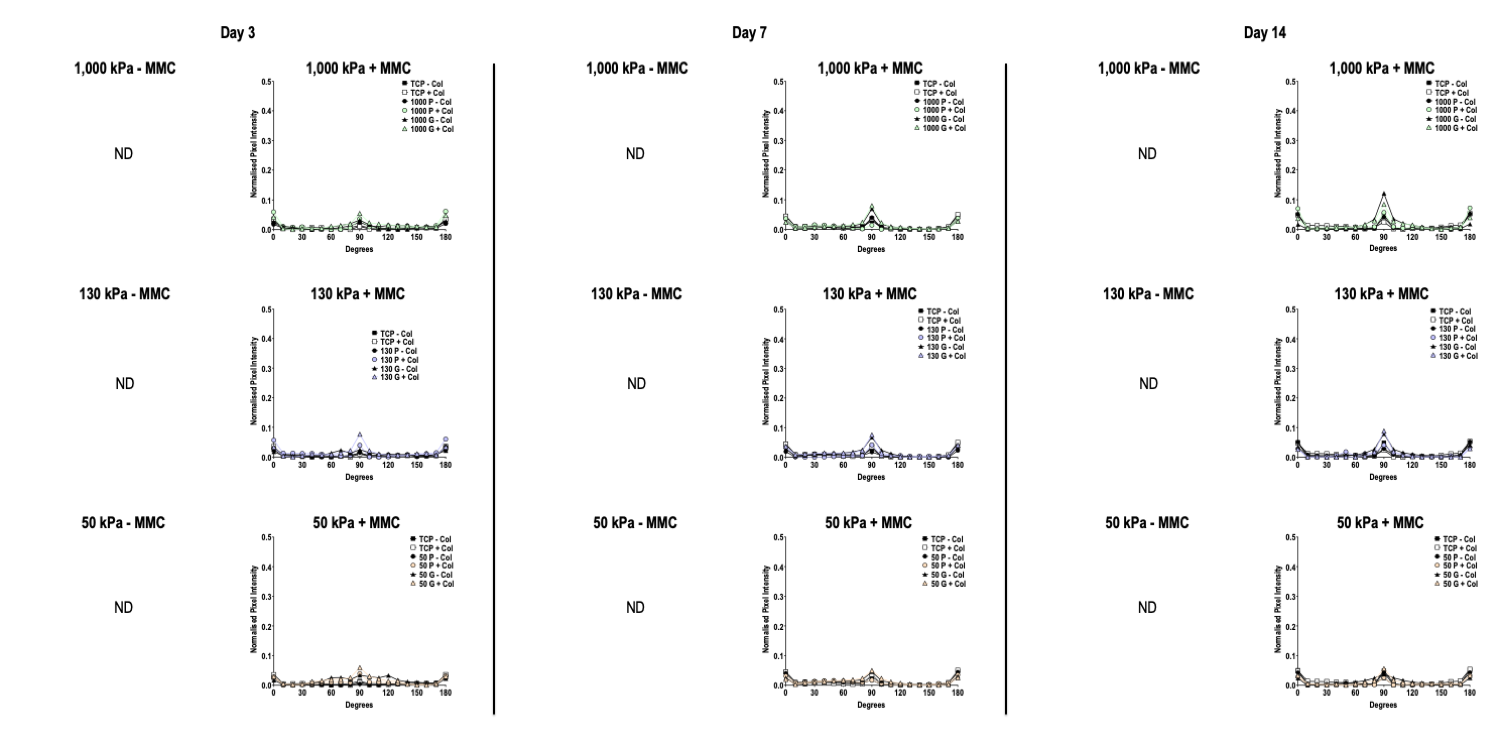


**Figure S29:** Human dermal fibroblast collagen type III matrix orientation at day 3, day 7 and day 14 on tissue culture plastic (TCP) without and with collagen type I coating (- Col, + Col) and macromolecular crowding (- MMC, + MMC) and on substrates of varying stiffness (1,000 kPa, 130 kPa, 50 kPa), surface topography [planar (P), grooved (G)], collagen type I coating (- Col, + Col) and macromolecular crowding (- MMC, + MMC). ND indicates conditions that did not produce sufficient matrix for analysis.


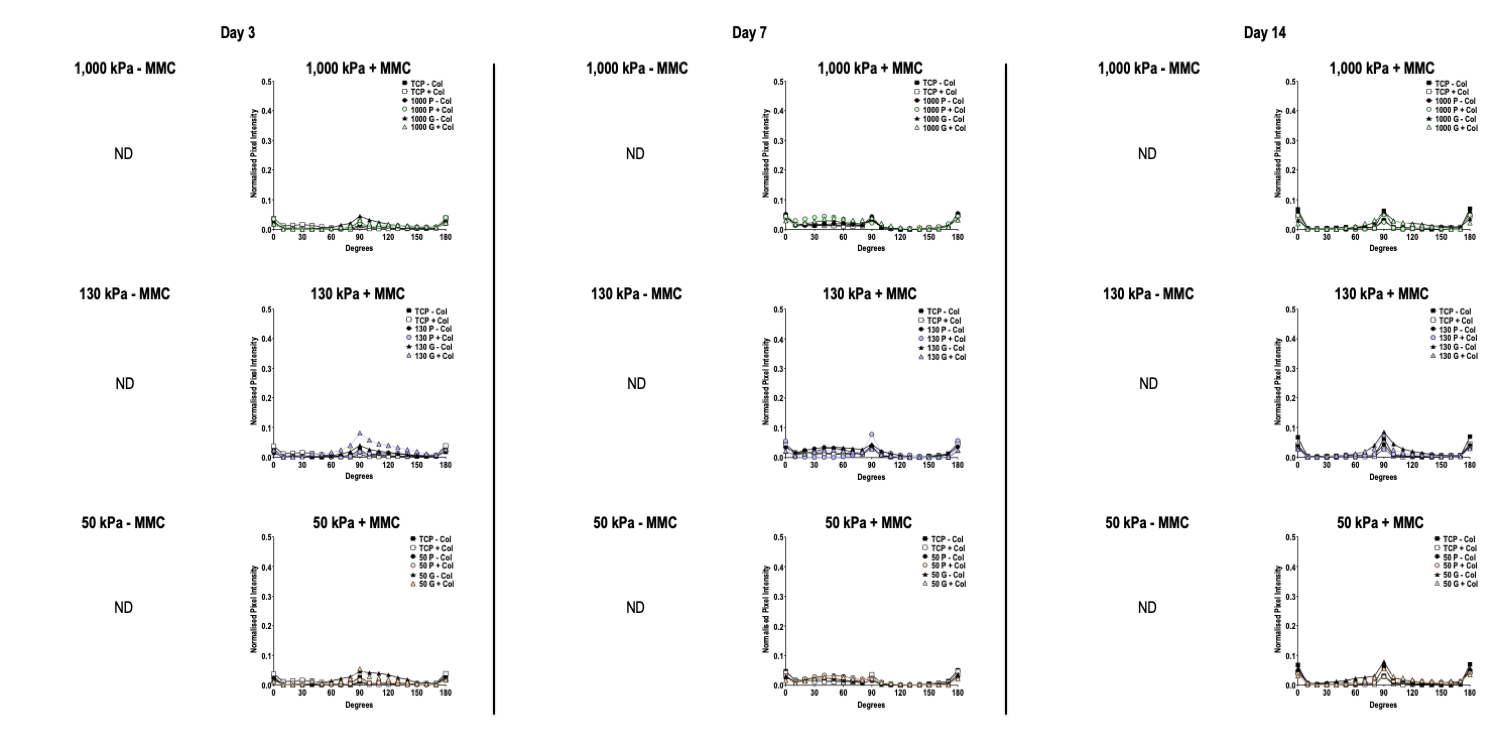


**Figure S30:** Human dermal fibroblast collagen type IV matrix orientation at day 3, day 7 and day 14 on tissue culture plastic (TCP) without and with collagen type I coating (- Col, + Col) and macromolecular crowding (- MMC, + MMC) and on substrates of varying stiffness (1,000 kPa, 130 kPa, 50 kPa), surface topography [planar (P), grooved (G)], collagen type I coating (- Col, + Col) and macromolecular crowding (- MMC, + MMC). ND indicates conditions that did not produce sufficient matrix for analysis.


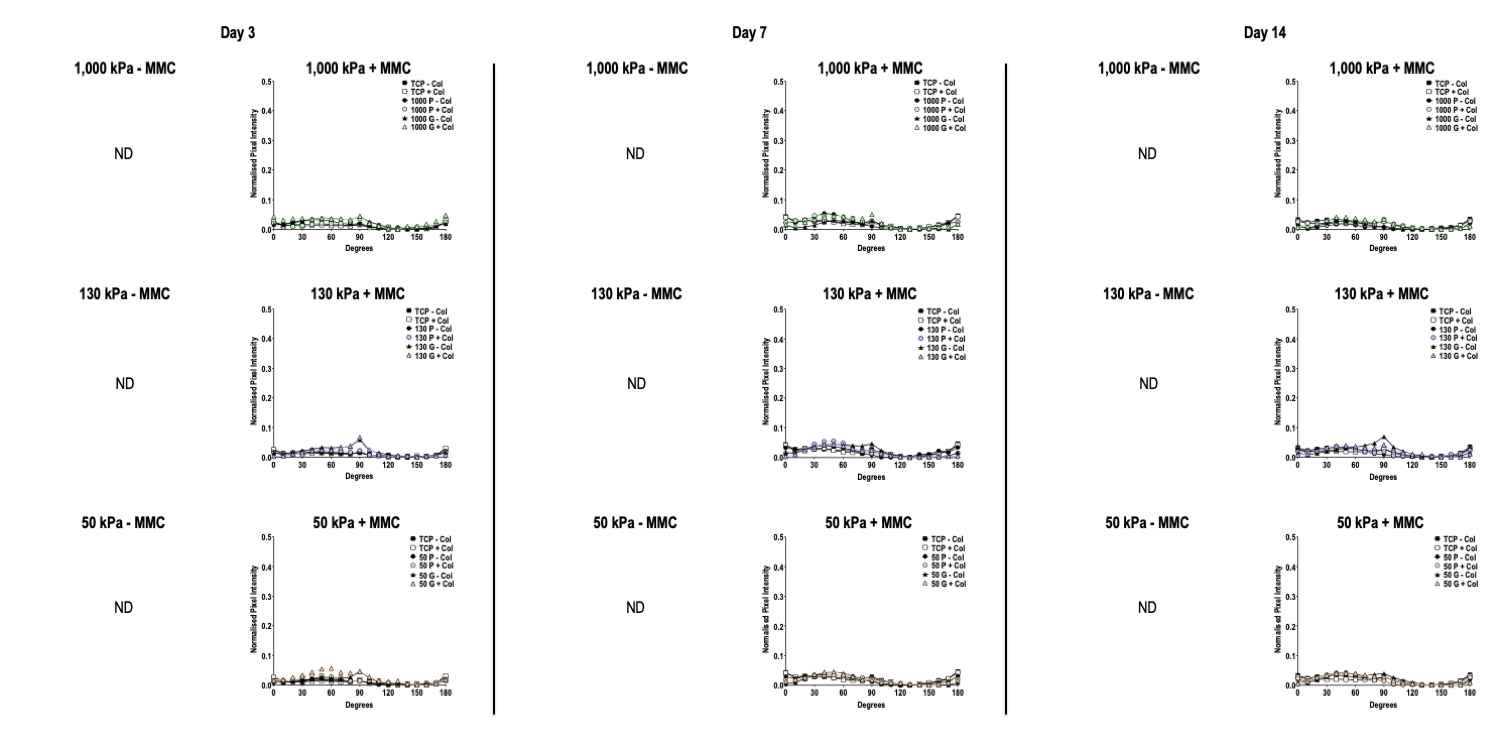


**Figure S31:** Human dermal fibroblast collagen type V matrix orientation at day 3, day 7 and day 14 on tissue culture plastic (TCP) without and with collagen type I coating (- Col, + Col) and macromolecular crowding (- MMC, + MMC) and on substrates of varying stiffness (1,000 kPa, 130 kPa, 50 kPa), surface topography [planar (P), grooved (G)], collagen type I coating (- Col, + Col) and macromolecular crowding (- MMC, + MMC).


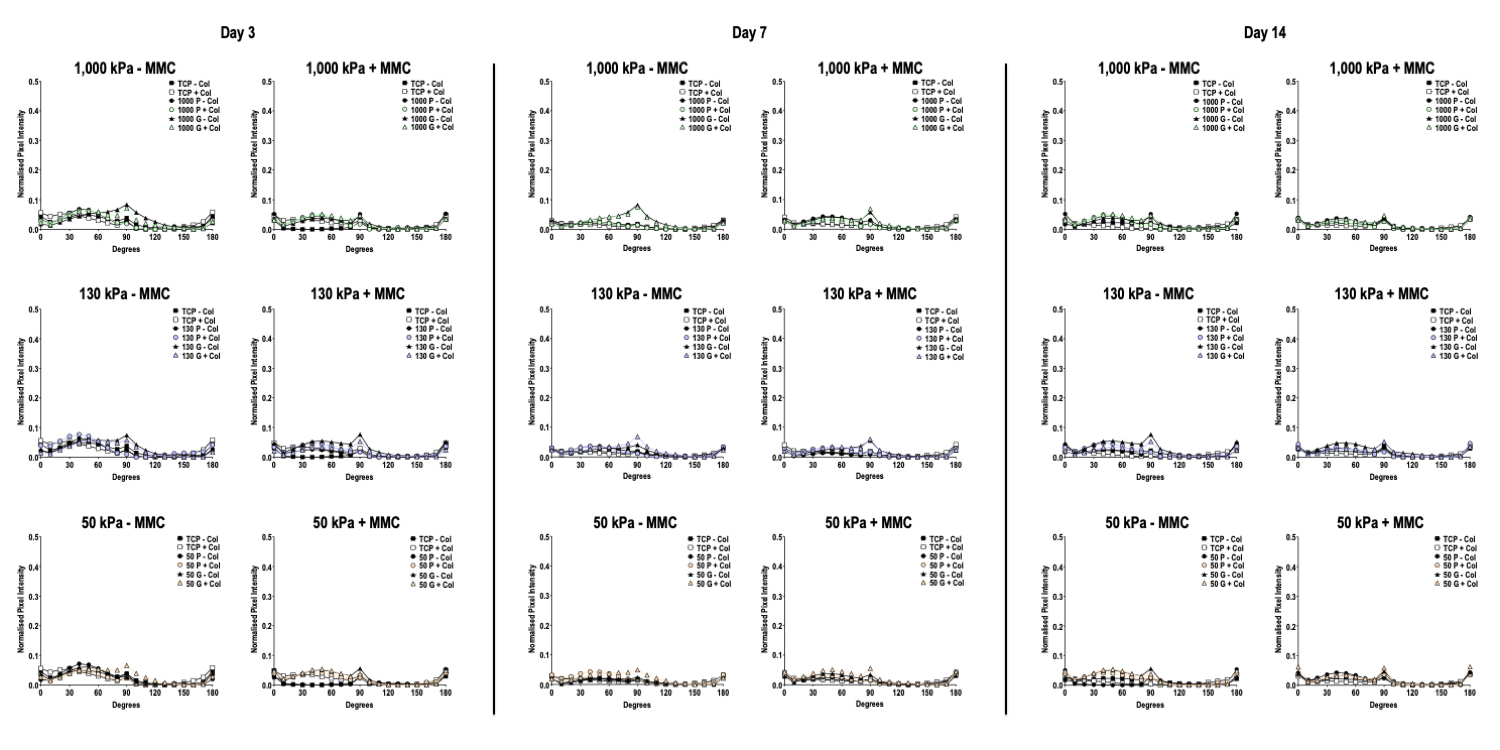


**Figure S32:** Human dermal fibroblast collagen type VI matrix orientation at day 3, day 7 and day 14 on tissue culture plastic (TCP) without and with collagen type I coating (- Col, + Col) and macromolecular crowding (- MMC, + MMC) and on substrates of varying stiffness (1,000 kPa, 130 kPa, 50 kPa), surface topography [planar (P), grooved (G)], collagen type I coating (- Col, + Col) and macromolecular crowding (- MMC, + MMC).


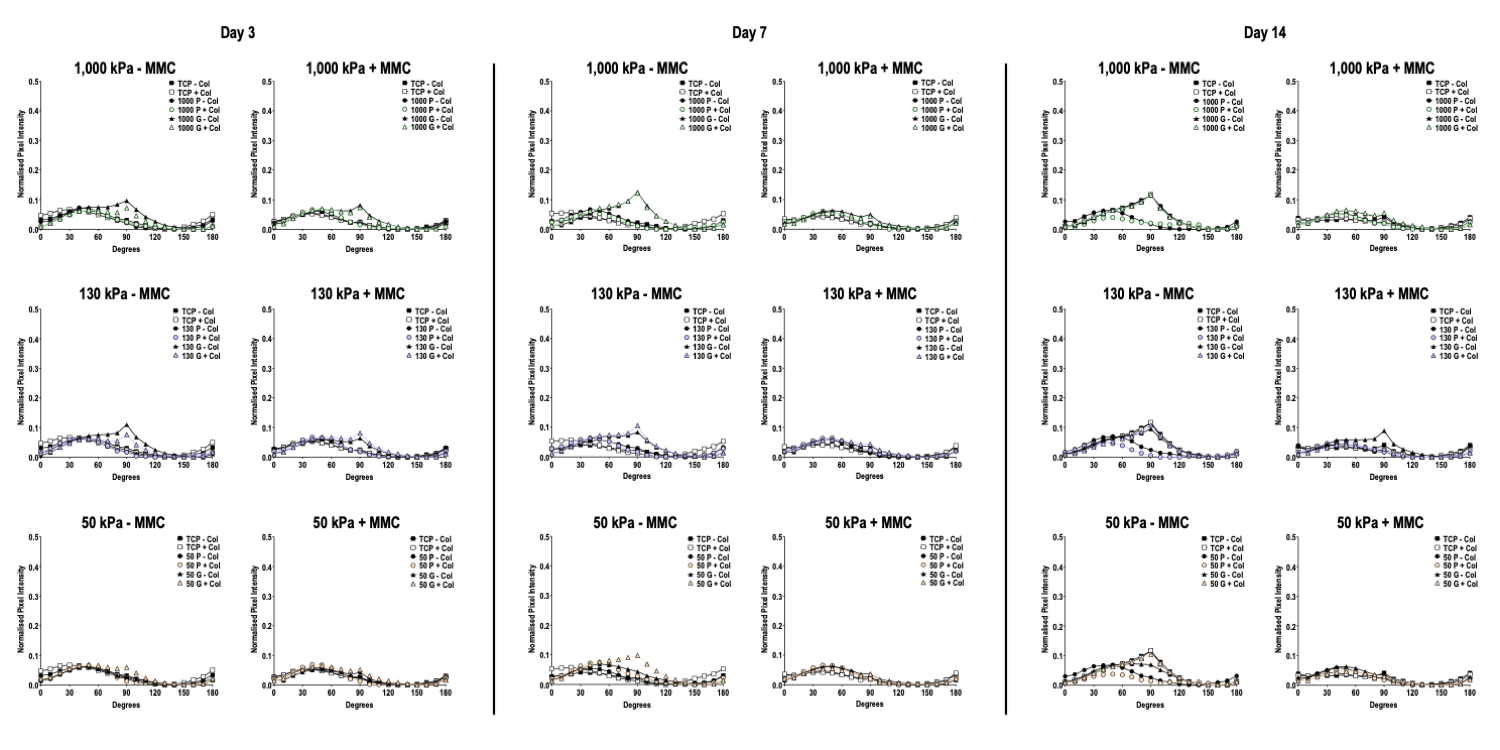


**Figure S33:** Human dermal fibroblast fibronectin matrix orientation at day 3, day 7 and day 14 on tissue culture plastic (TCP) without and with collagen type I coating (- Col, + Col) and macromolecular crowding (- MMC, + MMC) and on substrates of varying stiffness (1,000 kPa, 130 kPa, 50 kPa), surface topography [planar (P), grooved (G)], collagen type I coating (- Col, + Col) and macromolecular crowding (- MMC, + MMC).


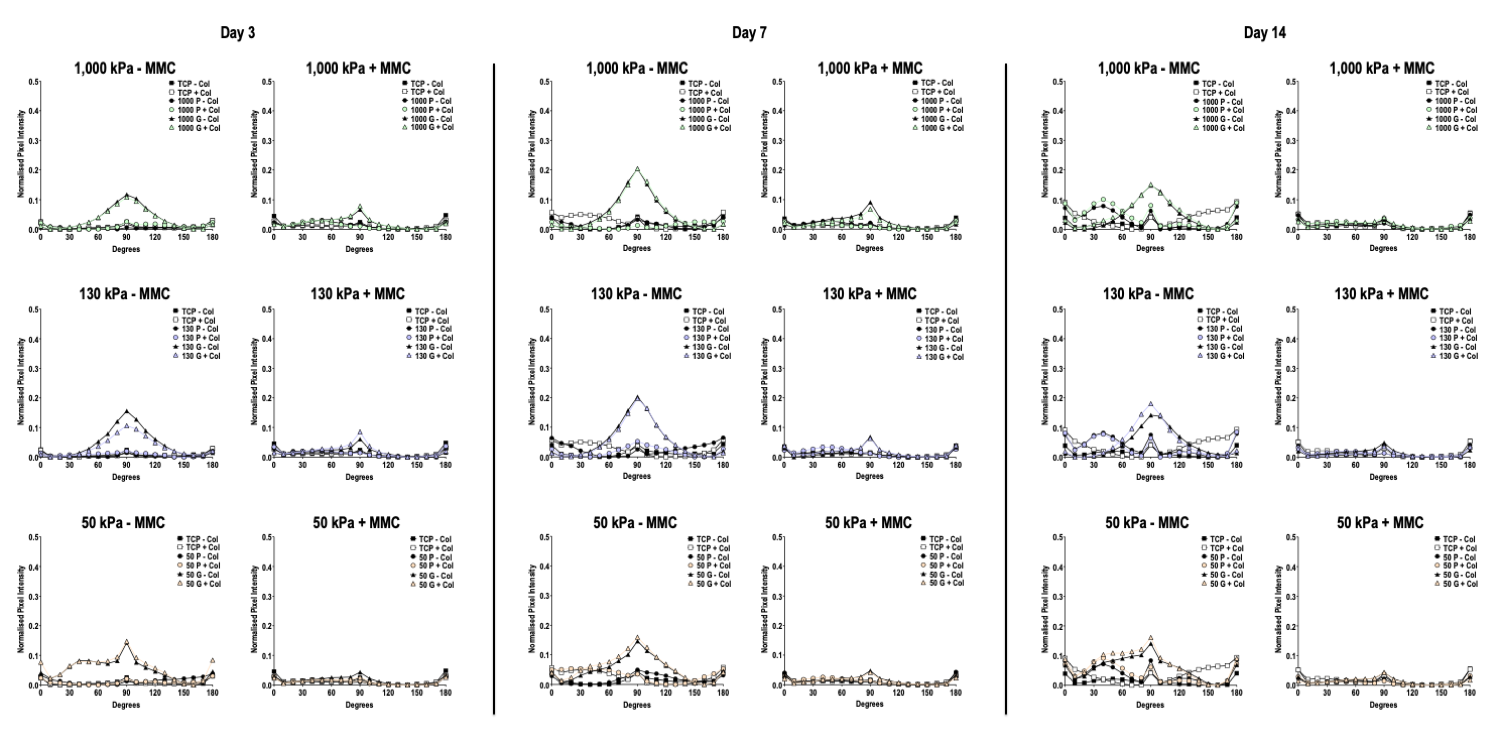

Supplement: Supplementary file 1 [file mmc1.docx]
